# Interactome analysis of Bag-1 isoforms reveals novel interaction partners in endoplasmic reticulum-associated degradation

**DOI:** 10.1371/journal.pone.0256640

**Published:** 2021-08-24

**Authors:** Nisan Denizce Can, Ezgi Basturk, Tugba Kizilboga, Izzet Mehmet Akcay, Baran Dingiloglu, Ozge Tatli, Sevilay Acar, Pelin Ozfiliz Kilbas, Efe Elbeyli, Serena Muratcioglu, Ayse Tarbin Jannuzzi, Attila Gursoy, Ozlem Keskin, Hamdi Levent Doganay, Betul Karademir Yilmaz, Gizem Dinler Doganay

**Affiliations:** 1 Department of Molecular Biology—Genetics and Biotechnology, Istanbul Technical University, Istanbul, Turkey; 2 Molecular Biology and Genetics Department, Istanbul Medeniyet University, Istanbul, Turkey; 3 Department of Molecular Biology and Genetics, Istanbul Kultur University, Istanbul, Turkey; 4 Department of Chemical and Biological Engineering, Koc University, Istanbul, Turkey; 5 Faculty of Pharmacy, Department of Pharmaceutical Toxicology, Istanbul University, Istanbul, Turkey; 6 GLAB, Umraniye Teaching and Research Hospital, Istanbul, Turkey; 7 Department of Biochemistry, School of Medicine/Genetic and Metabolic Diseases Research and Investigation Center, Marmara University, Istanbul, Turkey; University of Michigan, UNITED STATES

## Abstract

Bag-1 is a multifunctional protein that regulates Hsp70 chaperone activity, apoptosis, and proliferation. The three major Bag-1 isoforms have different subcellular localizations and partly non-overlapping functions. To identify the detailed interaction network of each isoform, we utilized mass spectrometry-based proteomics and found that interactomes of Bag-1 isoforms contained many common proteins, with variations in their abundances. Bag-1 interactomes were enriched with proteins involved in protein processing and degradation pathways. Novel interaction partners included VCP/p97; a transitional ER ATPase, Rad23B; a shuttling factor for ubiquitinated proteins, proteasome components, and ER-resident proteins, suggesting a role for Bag-1 also in ER-associated protein degradation (ERAD). Bag-1 pull-down from cells and tissues from breast cancer patients validated these interactions and showed cancer-related prominence. Using *in silico* predictions we detected hotspot residues of Bag-1. Mutations of these residues caused loss of binding to protein quality control elements and impaired proteasomal activity in MCF-7 cells. Following CD147 glycosylation pattern, we showed that Bag-1 downregulated VCP/p97-dependent ERAD. Overall, our data extends the interaction map of Bag-1, and broadens its role in protein homeostasis. Targeting the interaction surfaces revealed in this study might be an effective strategy in the treatment of cancer.

## Introduction

Bag-1 protein is a member of the Bcl-2-associated athanogene (BAG) protein family, which is evolutionary conserved in many organisms including yeast, plants and animals. Bag-1 functions as a co-chaperone for Hsp70/Hsc70 proteins [1,2]. In mammals Bag-1 has a pivotal role in the prevention of apoptotic cell death [3]. Bag-1 knockout (KO) mice die at embryonic stage due to the death of hematopoietic and neuronal progenitor cells [4]. Bag-1 is overexpressed in many types of cancer, including breast, prostate, and non-small cell lung cancers [5–7]. High expression levels of Bag-1 promote cell proliferation of cancer cells, and prevent drug-induced apoptosis [8–10]. In contrast, Bag-1 knockdown and knockout cells display reduced cell viability, decreased proliferation and increased sensitivity to anti-cancer drugs [11]. Given the well-established role of Bag-1 in cancer cell survival, it is a promising molecular target for the treatment of cancer [12].

Bag-1 exists in three isoforms in human cells: Bag-1L (52 kDa), Bag-1M (46 kDa), and Bag-1S (33 kDa). These isoforms are produced by alternative translation initiation from a single mRNA transcript [13]. Hence, they vary at their N-termini while invariably containing the evolutionarily conserved BAG and Ubiquitin-Like (UbL) domains at the C-termini [14]. Bag-1 is the nucleotide exchange factor (NEF) of Hsp70/Hsc70 molecular chaperones, and regulates folding of newly synthesized or misfolded proteins [15,16]. In addition to its function as a NEF, Bag-1 is also involved in the regulation of many important cellular pathways including regulation of apoptosis, proliferation, cell migration, and transcription [1,3].

The BAG domain of Bag-1 interacts with the ATPase domain of Hsp70, accelerating the release of ADP and client protein from Hsp70. BAG domain also binds to the MAP kinases C-Raf and B-Raf [17], Akt kinase [3], Bcl-2 [18], and nuclear hormone receptors [19]. Bag-1–Raf interaction is essential for phosphorylation of Bad protein and suppresses its proapoptotic function [3]. The UbL domain of Bag-1 is important for its binding to the 26S proteasome [20], and possibly to E3 ligases CHIP and SIAH [21,22]. In accordance with this, Bag-1 was shown to regulate proteasomal degradation of several proteins including Tau [23], BCR-ABL [20], and hERG [24].

Bag-1 isoforms vary in their subcellular localization due to their differential N-terminal regions. Bag-1L has a nuclear localization signal (NLS), and is a predominantly nuclear protein. Bag-1M and Bag-1S lack NLS and are mostly found in the cytoplasm, but they can be translocated to the nucleus under stress conditions [25]. Bag-1M and L isoforms have ten copies of the “TXSEEX repeats”, which are important for binding to DNA and transcriptional transactivation function, whereas Bag-1S has four copies [26]. Bag-1 isoforms show different binding affinities for certain interaction partners such as DNA and nuclear hormone receptors [27–30]. Moreover, they also have non-overlapping functions. Examples include the differential functional effects of Bag-1 isoforms on the transactivation activity of glucocorticoid receptor [31] and androgen receptor [32], and folding activity of Hsc70 [33].

Several interactors of Bag-1 were identified by standard molecular techniques in an isoform-independent manner, but detailed and isoform-specific interaction maps are necessary for elucidating the role of each Bag-1 isoform in critical cellular pathways. To fill this gap, we here identified interactome of Bag-1 isoforms from MCF-7 breast cancer cells using tandem affinity purification (TAP) followed by mass spectrometry (MS). Bag-1 isoform-specific complexes were also identified by blue native-PAGE (BN-PAGE) and subsequent LC-MS/MS analysis. Combination of these interactome-based studies revealed multiple Bag-1 complexes associated with various protein quality control (PQC) pathways. Co-immunoprecipitation assays from cell culture and tissues from breast cancer patients verified novel Bag-1 interactors including VCP/p97 and Rad23B. Computational approaches showed the surface residues at interaction sites of Bag-1 complexes that can be targeted for therapeutic purposes. Introduction of mutations on predicted hotspot residues experimentally confirmed the importance of these protein-protein interactions by altering the association with PQC network and proteasome machinery in cancer cells.

## Materials and methods

### Plasmids and antibodies

For Tandem Affinity Purification (TAP), a TAP tag, which consists of protein A and calmodulin binding peptide (CBP) separated by a Tobacco etch virus (TEV) cleavage site, was added before the start codon of each Bag-1 isoform, and cloned into pEZ-M02-plasmid vector (CS-L0198-M02; Capital Biosciences, MD, USA). Mock vector contained only the TAP cassette.

For inhibitor treatment experiments, His_6_-tagged Bag-1S vectors were used. To construct His_6_-Bag-1S vector a hexahistidine (His_6_) tag and a TEV cleavage site was added to the N-terminus of Bag-1S isoform, and cloned into pcDNA 3.1 vector, using NheI and XhoI restriction enzymes.

Q5 Site-Directed Mutagenesis Kit (New England BioLabs) was used to generate single amino acid substitution mutants of Bag-1S. Primers used are listed in S1 Table.

Primary antibodies mouse α-Bag-1, rabbit α-β-actin, rabbit α-BiP, rabbit α-Rad23B, rabbit α-Calnexin, rabbit α-Calreticulin, rabbit α-Hsp70, rabbit α-Hsp90, rabbit α-VCP/p97, rabbit α-ERp57 and rabbit α-Vinculin, and secondary antibodies HRP-conjugated α-rabbit IgG and α-mouse IgG were purchased from Cell Signaling Technology. Primary antibody mouse α-Emmprin was purchased from Santa Cruz.

### Cell culture

MCF-7 (ATCC^®^ HTB-22^™^) human breast cancer cells were grown in DMEM, high glucose, pyruvate (Gibco) supplemented with 10% fetal bovine serum (FBS), 100 units/ml penicillin and 100 μg/ml streptomycin (Thermo Fisher). MCF-12A (ATCC^®^ CRL-10782^™^) human breast epithelial cells were grown in DMEM/F12 (1:1) medium (Gibco), supplemented with 10% horse serum (Invitrogen), 100 units/ml penicillin and 100 μg/ml streptomycin (Thermo Fisher), 2.5 mg/ml insulin (Invitrogen), 150 μg/ml cholera enterotoxin (Sigma-Aldrich), 2.5 mg/ml hydrocortisone (Sigma-Aldrich) and 20 ng/ml epidermal growth factor (Sigma-Aldrich). Bag-1 knockout MCF-7 cells were generated using CRISPR-Cas9 system [9]. Cells were maintained at 37°C, 5% CO_2_ in a humidified incubator. 60–70% confluent cells were transfected with plasmids by using IN-fect *in vitro* transfection reagent (iNtRON Biotechnology) according to the manufacturer’s protocol. Cells were lysed 48 hours after plasmid transfection for protein purification or immunoblotting assays.

### Inhibitor treatment

5x10^5^ Bag-1 knock out MCF-7 cells per well were seeded into 6-well plates. 24 hour later, cells were transfected with mock vector, wild-type His- or TAP-tagged Bag-1S or mutants using PEI 25K transfection reagent (Polysciences) with a 1:3 DNA:PEI ratio. Inhibitor treatments were performed at 24 hours post-transfection. For tunicamycin treatment, cells were incubated with 10 μg/ml tunicamycin (AppliChem) for 24 hours. For emetine or CB-5083 treatment, transfected cells were incubated with 25 μM emetine (Cayman) or CB-5083 (MedChemExpress) for 4 hours. DMSO was used as negative control. Cells were harvested and lysed using Mammalian Cell Extraction kit (BioVision). Protein concentration was measured by Bradford (Bio-Rad) assay and equal amounts of cell lysates were analyzed by western blotting.

### MTT assay

1x10^4^ cells per well were seeded in 96-well plates. Following transfection with TAP–Bag-1 plasmids, they were maintained for 24, 48 and 72 hours prior to treatment with MTT reagent (Sigma-Aldrich). Colorimetric measurements were done at 570 nm and 655 nm, using Benchmark Plus ELISA microplate reader (Bio-Rad).

### Colony formation assay

Cells were seeded at 3x10^3^ cells per well in 6-well plates, and maintained for 10 days after transfection with TAP–Bag-1 plasmids. Following fixation in methanol/acetic acid (3:1), cells were stained with 0.5% crystal violet in methanol for 15 min, and washed with distilled water. Stained colonies were photographed with a digital camera.

### Affinity purification and in-solution tryptic digestion

Protein isolation from TAP–Bag-1 isoform-transfected cells was done by using Mammalian Cell Extraction Kit (BioVision). Protein concentration was measured with Bradford (Bio-Rad) assay. 500 μg proteins were incubated with IgG Sepharose 6 Fast Flow beads (GE Healthcare) overnight at 4°C. After a short centrifugation, supernatants were collected as unbound proteins and the pellets were treated with AcTEV protease (Thermo Fischer). Beads were centrifuged, and the supernatants containing Bag-1 isoform-bound proteins were collected. For the immunoblotting analyses, 20 μg total protein extract and 1:5 of the total volume of unbound and bound proteins were loaded on polyacrylamide gel. For the LC-MS/MS analyses, 20 μg purified proteins were first treated with 8 M urea for 15 min, and then sequentially with 5 mM DTT, 25 mM iodoacetamide (IAD), and 5 mM DTT solutions at room temperature for 60 min. Proteins were overnight digested by 1 μg trypsin (Sigma-Aldrich) at 37°C. Trypsin activity was stopped by formic acid, and peptide mixture was stored at -20°C.

### Blue Native-Polyacrylamide Gel Electrophoresis (BN-PAGE) and in-gel tryptic digestion

Protein extraction from transfected cells was performed by using NativePAGE Sample Buffer (Thermo Scientific) supplemented with 5% digitonin to conserve their native structures. Bag-1 isoform-specific complexes were purified by affinity purification as described above. BN-PAGE was performed using XCell SureLock™ Mini-Cell Electrophoresis System (GE Healthcare) by following the procedure described in *Wittig et al*. *2006* [34]. After electrophoresis, gel was fixed in 40% methanol, 10% acetic acid for 30 min. Stained bands were cut and destained in 1:1 acetonitrile (ACN): 100 mM ammonium bicarbonate (ABC). Gel pieces were reduced in 2mM TCEP, 50 mM ABC and alkylated in 12 mM IAD, 50 mM ABC in dark. After washing in 1:3 ACN:50 mM ABC, gel pieces were dehydrated in ACN and dried in vacuum concentrator. 20 μl of 20 ng/μL trypsin was added, and incubated overnight at 37°C. Peptide mixtures were dissolved in 1% formic acid and 100 mM ABC, and stored at -20°C until LC-MS/MS analysis.

### Peptide mapping via LC-MS/MS

Identification of digested peptides via mass spectrometry was performed by using Acquity UPLC coupled with Waters Synapt G2-Si HDMS (Waters Corp). Mobile phase A was water, mobile phase B was acetonitrile (ACN), and mobile phase C was water containing 1% (v/v) formic acid. Digested samples were loaded on BEH C18 RP analytical column. Separation of peptides was accomplished with a gradient of 1–42% mobile phase B over 60 min followed by a quick ramp from 42% to 80% in 1 min, and isocratic elution at 80% for 3 min. Re-equilibration of column was carried out over 20 min. Mobile phase C and flow rate were kept constant at 10% and 200 μL/min, respectively, during the run, and the column temperature was maintained at 65°C.

All analyses were performed in positive electrospray ionization and resolution mode. In low energy mode, collision energy was set at 4 V, while the elevated collision energy was ramped from 25V to 40 V. The source temperature and the capillary voltage were set to 120°C and 3 kV, respectively. Glu-fibrinopeptide B was infused to calibrate time-of-flight analyzer from 50 to 2000 m/z. It was also used as internal calibrant in reference spray with a frequency of 30 s^-1^.

### MS data analysis

Raw data files were processed using Progenesis QI (v4.1; Nonlinear Dynamics). UNIPROT Homo sapiens taxonomy was used as the searching database. Database search was limited to fully tryptic peptides with a maximum of one missed cleavage. False discovery rate was set to <1%. Carbamidomethyl (C) was defined as fixed modification while Oxidation (M), Acetylation (N-term), Deamidation (N,Q) and Phosphorylation (S,T,Y) were included as variable modifications. Ion matching parameters were set as ≥ 3, 7, 2 for fragments/peptide, fragments/protein and peptides/protein, respectively. Proteins were quantified using label-free relative quantification based on abundances of non-conflicting peptides. Fold changes were calculated from quantification of three replicates of treatment samples relative to control samples. Gene ontology (GO) enrichment and KEGG pathways were analyzed for identified interaction partners of Bag-1 isoforms using DAVID functional annotation tool [35]. Heatmap for enriched proteins in Bag-1 isoform-specific interactomes was drawn using GraphPad Prism (v8.0.1; GraphPad Software).

### Co-immunoprecipitation assay for protein extracts from cell lines and tissues

For co-IP assays from untransfected cell lines we used Mammalian Cell Extraction Kit (BioVision) as mentioned above.

For co-IP assays from breast tissues, samples were obtained from 12 female breast cancer patients registered in Umraniye Teaching and Research Hospital (UEAH) in Istanbul, as described in *Kizilboga et al*. *2019* [9]. Informed consent was obtained from all subjects involved in the study, which was conducted according to the guidelines of the Declaration of Helsinki, and approved by the Ethics Committee of University of Health Sciences Istanbul Umraniye Teaching and Research Hospital (UEAH), protocol code BD8082998622/4864. To examine the interaction of Bag-1 with VCP/p97, Rad23B and Hsp70 in the tumor and neighboring normal tissues, frozen tissues were ground using pestle and mortar in liquid nitrogen, and suspended in T-PER tissue protein extraction reagent (Thermo Scientific), supplemented with 1x PhosSTOP (Roche) and 1x cOmplete Protease Inhibitor Cocktail (Roche). Homogenates were centrifuged at 10000 *g*, 4°C for 5 min, and the supernatants were collected as tissue lysates. To reduce any non-specific binding and background that can be caused by Human IgG, lysates were pre-cleaned by using protein A/G beads (Pierce, Thermo Scientific). Protein concentration was measured with Bradford assay.

Dynabeads Protein G (Invitrogen) were incubated with monoclonal α-Bag-1 antibody for 30 min. 250 μg total protein extracts were overnight incubated with both antibody-coupled and uncoupled beads at 4°C with rotation to verify the lack of unspecific binding of putative interaction partners to the used matrix. Supernatants were collected as unbound, and immunoprecipitates were eluted in 20 μl elution buffer (50 mM glycine, pH 2.8).

### Immunoblotting analysis

Proteins were denatured by boiling in Laemmli buffer at 95°C for 5 min, separated on 12% SDS-PAGE, and transferred to a nitrocellulose membrane using iBlot Turbo transfer system (Invitrogen) at 20 V for 7 minutes. Membranes were blocked in 5% non-fat dry milk in TBS containing 0.1% Tween20 (TBS-T). Primary antibodies (1:500) were treated overnight at 4°C. Membranes were washed with TBS-T and incubated with secondary antibodies (1:5000) for 2 hours. After the final wash step, membranes were treated with ECL substrate and visualized in ChemiDoc MP imaging system (Bio-Rad). Densitometric analysis was performed using ImageLab (Bio-Rad).

### Immunocytochemistry

2.5x10^4^ Bag-1 KO MCF-7 cells per well were seeded in 12-well plates containing poly-L-lysine coated 20 mm coverslips. 48 hours after transfection with TAP–Bag-1 isoforms, cells were washed with phosphate buffered saline (PBS) solution, and fixed in 4% formaldehyde for 15 min at -20°C. Following blocking in 3% BSA in PBS, cells were overnight incubated with primary antibodies (1:200) at 4°C. After three washing steps, cells were incubated with Alexa Flour® 647 goat α-mouse and Alexa Flour® 488 goat α-rabbit secondary antibodies (Invitrogen, 1:1000 for both) at 37°C for 1 hour. After the final wash, coverslips were incubated with ER-Tracker™ Blue-White DPX dye (Thermo Scientific) and mounted on slides using mounting medium (Sigma). Confocal images were obtained under 40x magnification using Leica DMi8 imaging system.

### Computational prediction of Bag-1 complex structures

Structures of Bag-1 complexes with selected proteins were modelled using PRISM [36,37] and RosettaDock [38]. Structures of unbound target proteins were obtained from Protein Data Bank (PDB). 1 structure for the UbL domain (residues 29–109) of Bag-1 and 3 structures with a resolution higher than 2.0 Å for the BAG domain (residues 131–211) were used (S2 Table). To avoid redundancy due to multiple structures per protein, all available structures of the same protein were superimposed, and RMSDs were calculated. The RMSD threshold was determined considering the size of the protein (0.5 Å or 0.8 Å). A representative from each cluster was chosen among X-ray/NMR structures with the highest resolution (S2 Table), which reduced the number of total target structures from 330 to 50. Calnexin, and UBA1 and UBA2 domains of Rad23B, which do not have a crystal structure, were modelled using I-TASSER [39]. Complex structures predicted by PRISM with a binding energy score (BES) of -10 or lower, and by RosettaDock with an interface score (I_sc) in the range between -3.5 and -10 were selected. These complex structures were visualized by using PyMOL Molecular Graphics System (v2.0; Schrödinger, LLC) and interacting residues were determined by using BIOVIA Discovery Studio Visualizer (v19.1.0.18287; Dassault Systemès). The residues at protein interfaces, mutations of which change the binding free energy at least 2.0 kcal/mol, were identified as hotspot residues by the Hotregion database [40]. Effects of single amino acid substitutions in the UbL and BAG domains on interactions between Bag-1 and Hsp70, VCP/p97, and Rad23B were analyzed by FoldX [41,42] and ΔΔG (kcal/mole) values were classified as in *Studer et al*, *2014* [43].

### Proteasomal activity assay

Pellets of 1x10^6^ cells were lysed in lysis buffer (0.25 M saccharose, 25 mM Hepes, 10 mM MgCl_2_, 1 mM EDTA and 1 mM DTT, pH 7.4) by three freeze-thaw cycles, and centrifuged at 14000 *g*, 4°C for 30 min. Supernatants were transferred into new tubes, and incubated with 200 μM fluorogenic peptide substrate Suc-LLVY-MCA (Sigma-Aldrich) at 37°C for 30 min. Fluorescence of released MCA was measured at 460 nm with excitation at 360 nm. Total (20S and 26S) proteasomal activity was measured after adding 2 mM ATP (Thermo Fisher Scientific). 20S proteasomal activity was measured in ATP-depleted conditions maintained by adding 5 U hexokinase (Roche Diagnostics) and 15 mM 2-Deoxy-D-glucose (Glentham Life Sciences). BCA assay was performed to measure protein concentrations and activities were calculated as MCA concentration/(mg protein x min). Data were indicated as relative proteasome activities in Fig 6.

### Statistical analysis

All experiments were performed in triplicates unless otherwise specified. Unpaired t-test assuming unequal variances, or one-way or two-way ANOVA tests were used to determine statistical significances (*p*<0.05) in GraphPad Prism 8.0.1. *p*-values were represented as following: *p<0.05; **p<0.01, ***p<0.001, and ****p<0.0001.

## Results

### Isoform-specific Bag-1 interactome analysis by TAP/MS

**Fig 1:**
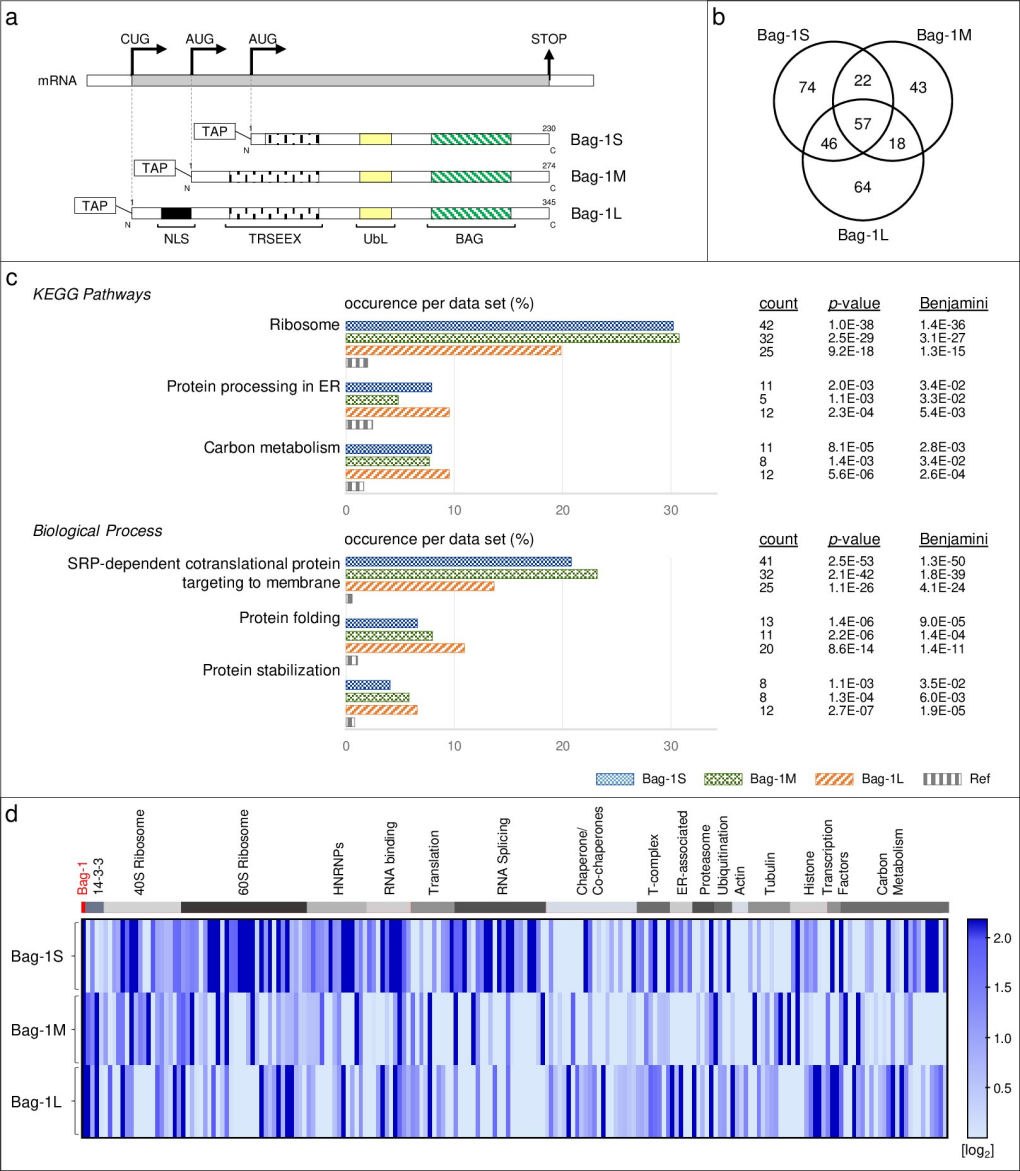
Bag-1 isoform-specific interactome analysis and classification of enriched pathways. **a**. Schematic representation of TAP–Bag-1 constructs used in the interactome analysis. TAP: Tandem Affinity Purification, NLS: Nuclear localization signal, TRSEEX: Amino acid repeats, UbL: Ubiquitin-like domain, BAG: BAG domain. **b**. Venn diagram of candidate interaction partners enriched (>1.2 fold) in the Bag-1 isoforms’ interactomes relative to the mock interactome. **c**. Subset of Gene Ontology (GO) classifiers for candidate Bag-1-interactors. Percentage of proteins with the indicated GO terms within Bag-1 interactomes and the reference dataset are depicted. Blue: Bag-1S, green: Bag-1M, purple: Bag-1L and grey: Reference. **d**. Heatmap analysis for the relative abundances of the binding partners for each Bag-1 isoform. Enriched proteins are clustered according to their biological functions. Color code is given in log_2_ base.

To study the isoform-specific interactome of Bag-1, we constructed vectors carrying TAP-tagged Bag-1S, M, and L isoforms (Fig 1A). The TAP tag was added at the N-terminus of each isoform because we previously observed that heterologous expression of C-terminal TAP-tagged Bag-1L also led to the expression of smaller TAP-tagged isoforms (S and M) due to the intrinsic ability of Bag-1 mRNA to initiate translation at alternative sites. Overexpression of all TAP–Bag-1 isoforms increased cell viability and proliferative capacity of MCF-7 breast cancer cells and MCF-12A non-tumorigenic breast epithelial cells, indicating that tagged Bag-1 isoforms were functional (S1 Fig).

For the interactome analysis we used MCF-7 cells, reasoning that interactions identified in these cells might give insights into the role of Bag-1 isoforms in cancer. We transfected MCF-7 cells with the vectors encoding each isoform and the mock vector. Bag-1 complexes were purified from cell lysates by TAP-tag affinity purification, digested by trypsin, and the resulting peptides were identified by LC-MS/MS. Relative quantification of proteins was done based on ion chromatogram intensities of peptides. We searched for proteins with >1.2-fold enrichment in Bag-1 isoform interactomes relative to the mock interactome, and found 199, 140 and 185 enriched proteins in the interactomes of Bag-1S, M and L, respectively (S3 Table). Out of all retrieved interaction partners (324 proteins in total), 57 proteins (18%) were shared in three isoforms, 86 proteins (27%) were shared in any two isoforms, and 181 proteins (56%) were unique to only one isoform (Fig 1B). Gene ontology (GO) enrichment analysis for these proteins showed largely overlapping functions for Bag-1 isoforms interactomes in KEGG pathways and biological processes. Enriched entities included proteins involved in ribosomes, protein processing in the ER, carbon metabolism, SRP-dependent co-translational protein targeting to the cell membrane, and protein folding and stabilization (Fig 1C).

Despite their qualitative similarities, Bag-1 isoform interactomes varied in a quantitative fashion. For instance, ribosomal proteins and RNA binding proteins were highly enriched in Bag-1S interactome, whereas histone proteins and transcription factors were highly enriched in Bag-1L interactome (Fig 1D). These variations may be due to differences in binding affinities and/or cellular localization of Bag-1 isoforms. Overall, the interactome analysis showed significant association of Bag-1 isoforms with protein processing and protein homeostasis, consistent with the co-chaperone function of Bag-1.

### Isoform specific Bag-1 complexes are enriched with PQC elements

**Fig 2:**
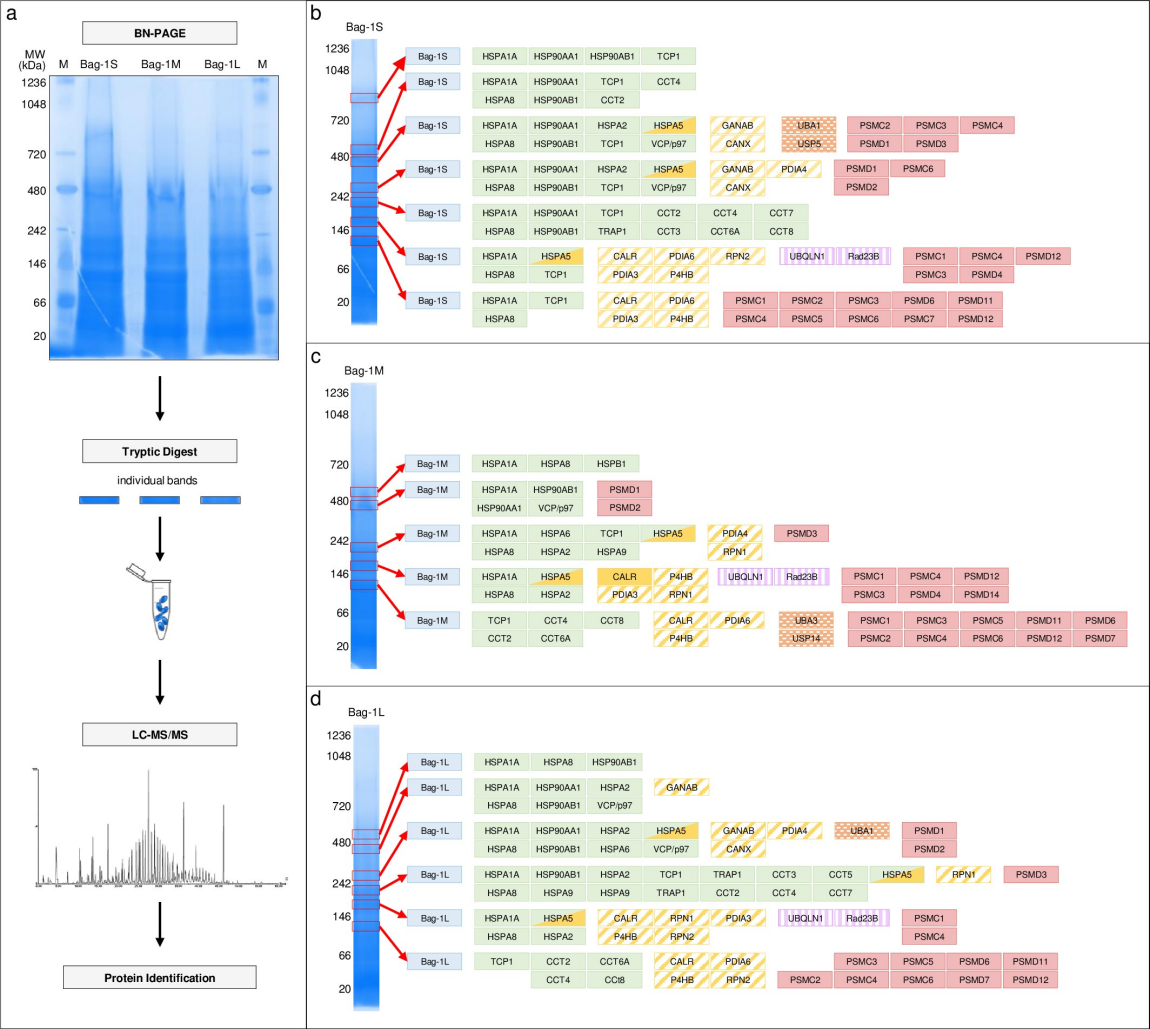
Components of isoform-specific Bag-1 complexes identified by BN PAGE/LC-MS/MS. **a**. Experimental workflow. Bag-1 immunoprecipitates were run on native gel. Individual bands in each lane were cut, trypsinized and the resulting peptides were analyzed by LC-MS/MS. M: Protein marker. **b-d**. Seven protein complexes were characterized for Bag-1S (**b**), five for Bag-1M (**c**), and six for Bag-1L (**d**). Identified proteins are color-coded as following: Blue: Bag-1, green: Chaperones, yellow: ER-resident proteins, orange: Ubiquitination elements, purple: Shuttling proteins, red: Proteasome subunits.

The co-isolation of several Bag-1 interactors with similar functions suggested that Bag-1 might be associated with large protein complexes. Putative Bag-1 complexes were further investigated by BN-PAGE. The bands in each lane were cut, subjected to in-gel tryptic digestion, and the resulting peptides were identified by LC-MS/MS (Fig 2A, S4 Table). Seven complexes were characterized for Bag-1S (Fig 2B), five for Bag-1M (Fig 2C), and six for Bag-1L (Fig 2D). BN-PAGE/MS analysis detected approximately half of the enriched interacting partners identified by TAP-MS (46.7%, 36.4% and 47.0% for Bag-1S, M and L, respectively). All Bag-1 complexes contained chaperone proteins. 26S proteasome regulatory subunits, the transitional ER ATPase VCP/p97, and the ER-resident chaperones protein disulfide isomerases (PDI), ribophorin, calreticulin and calnexin were also found in these complexes. The co-existence of ER-resident proteins with proteasome components and UbL-containing proteins, such as Rad23B and ubiquilin-1, in Bag-1 complexes was surprising, and suggested a link between Bag-1 and ERAD.

### Validation of novel interactors of Bag-1 isoforms in breast cell lines and tissues

**Fig 3:**
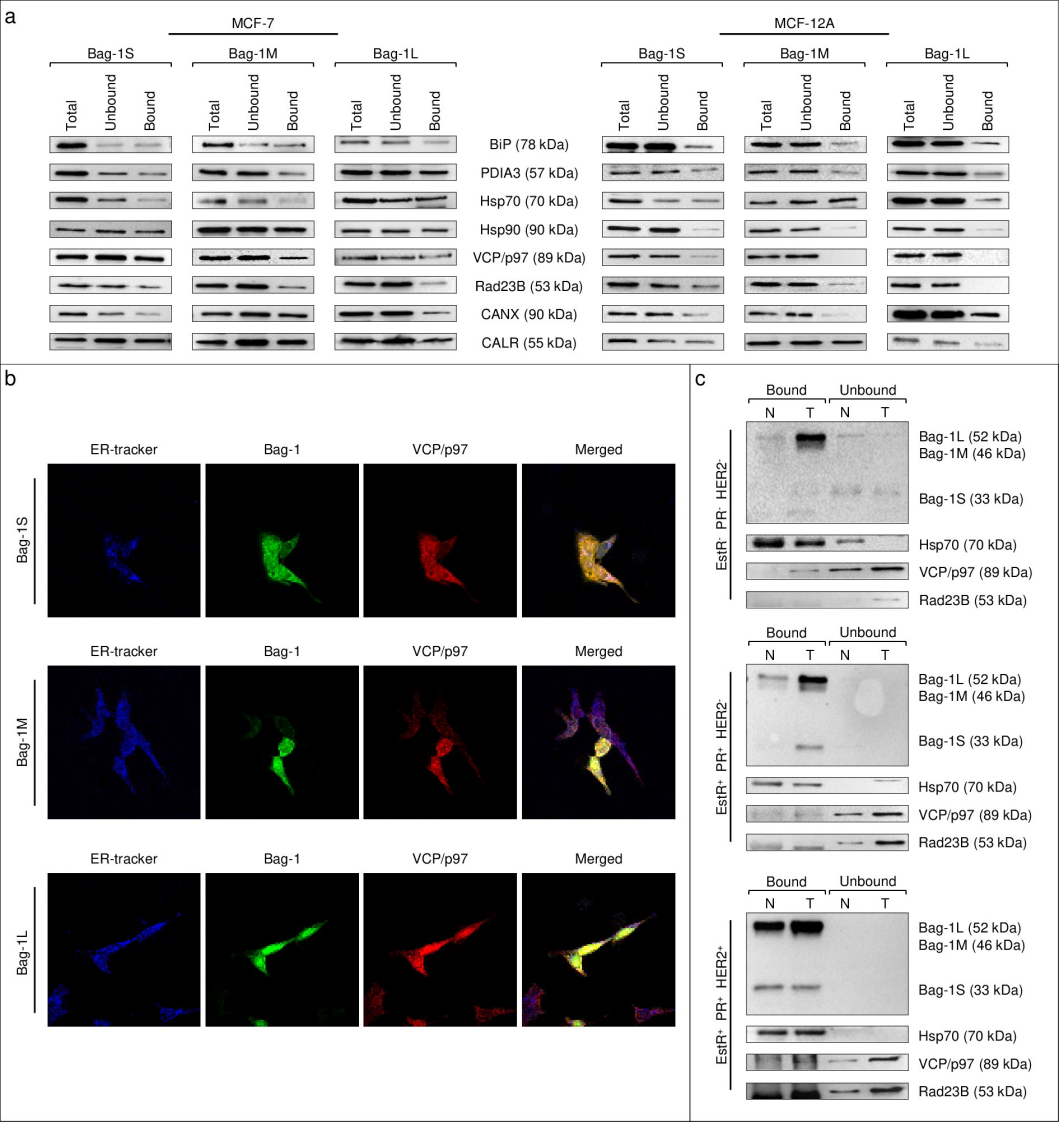
Bag-1 isoforms interact with protein quality control elements in breast cell lines and tissues. **a**. Immunoblotting assays for interaction partners of purified Bag-1S, M and L proteins extracted from MCF-7 and MCF-12A. Total, unbound, and Bag-1-bound protein extracts were blotted for BiP, PDIA3, Hsp70, Hsp90, VCP/p97, Rad23B, Calnexin and Calreticulin. **b**. Immunocytochemistry for assessment of Bag-1’s co-localization with interaction partners. MCF-7 cells transfected with Bag-1 isoforms were stained with Bag-1 antibody and VCP/p97. **c**. Co-immunoprecipitation assay in tissues from breast cancer patients. Interaction of Bag-1 with VCP/p97, Rad23B, and Hsp70 was increased in tumor tissues compared to normal. n = 4 for each molecular subtype of breast cancer. T: Tumor tissue, N: Neighboring normal tissue.

We further examined the putative interactions of Bag-1 with Hsp90, VCP/p97, Rad23B, PDIA3, calnexin and calreticulin, as well as its known interactions with Hsp70 and BiP, in MCF-7 and MCF-12A cells. Immunoblotting assays after TAP purification identified these interactions for all Bag-1 isoforms in MCF-7 cells, whereas VCP/p97, Rad23B and calnexin showed isoform-specific preferences in MCF-12A cells. Additionally, the interactions of Bag-1 isoforms with Hsp90, VCP/p97, Rad23B and calreticulin were more prominent in MCF-7 cells compared to MCF-12A cells (Figs 3A and S2). These interactions were not detected in untransfected cells proving that bindings were specific to TAP-Bag-1 constructs but not due to affinity matrices (S3 Fig). Lastly, interaction of Bag-1 with VCP/p97, calnexin, Rad23B and Hsp70 were also assessed by co-IP assays from untransfected MCF-7 and MCF12A cells. These assays confirmed higher interaction levels of Bag-1 with VCP/p97 and calnexin in MCF-7 cells compared to MCF-12A cells (S4 Fig).

Next, the cellular localization of Bag-1 and its interaction partners were analyzed by staining with the relevant antibodies in Bag-1 isoform-transfected Bag-1 KO MCF-7 cells (Fig 3B) and in untransfected wild-type MCF-7 cells (S5 Fig). Bag-1 staining was predominantly nuclear in Bag-1L transfected cells, whereas predominantly cytoplasmic in Bag-1S and Bag-1M-transfected cells. Co-localization of Bag-1 with Hsp70, VCP/p97 and Rad23B (all of which were both nuclear and cytoplasmic) was evident in the subcellular compartment where the Bag-1 isoform was mostly expressed.

To assess whether observed differences among MCF-7 and MCF-12A cells reflect cancer-related alterations, we examined protein-protein interactions involving Bag-1 in normal and tumor tissues from breast cancer patients. Tumor tissue samples were classified into three molecular subtypes according to their expression of nuclear hormone receptors (estrogen receptor (EstR), progesterone receptor (PR), and epidermal growth factor receptor (HER2)) as EstR^-^ PR^-^ HER2^-^, EstR^+^ PR^+^ HER2^-^, and EstR^+^ PR^+^ HER2^+^. Despite variable expression levels of the target proteins in tissues, co-IP assays using anti-Bag-1 antibody confirmed the interaction between Bag-1 and Hsp70, VCP/p97 and Rad23B in the majority of breast tissues regardless of molecular subtypes (Figs 3C and S6). Importantly, these interactions were more prominent in tumor tissues compared to normal tissues, possibly due to increased expression of the proteins in tumor cells.

### Structural predictions of Bag-1 complexes

The interactions of Bag-1 with important components of PQC systems represent potential drug targets. To gain structural insights into interactions between Bag-1 (PDB ID: 1HX1) and its putative binding partners, we modelled the complex structures with a template-based protein-protein interaction prediction tool, Protein Interactions by Structural Matching (PRISM) [36,37], using the structures available in Protein Data Bank (PDB) as targets (S2 Table).

**Fig 4:**
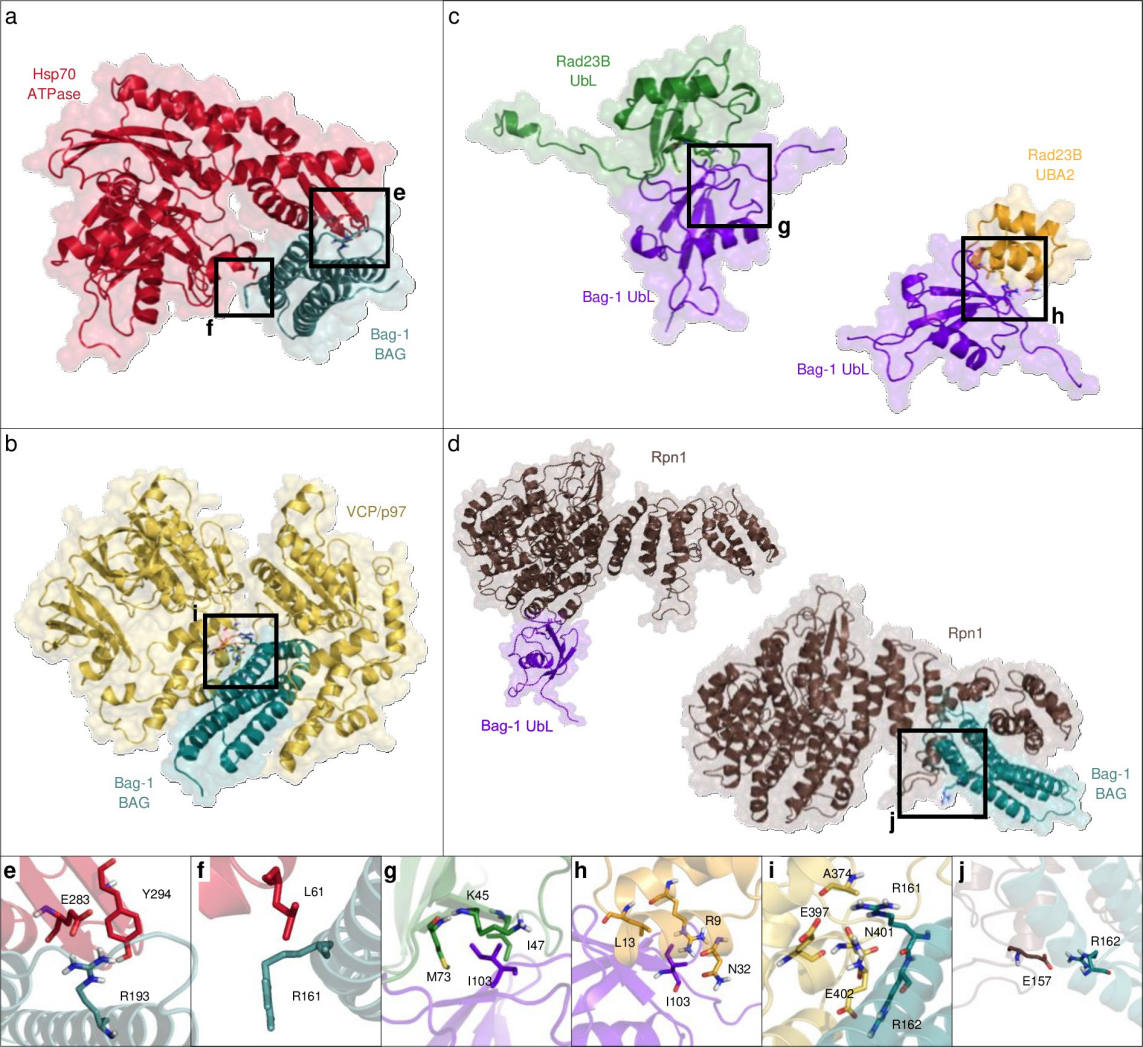
Structural predictions for Bag-1 complexes. Interaction of BAG domain of Bag-1 (turquoise) with **a**. the ATPase domain of Hsp70 (red), and **b**. the monomeric VCP/p97 protein (yellow). Interaction of UbL domain of Bag-1 (purple) with **c**. the UbL domain (green) and UBA2 domain (orange) of Rad23B. **d**. Interaction of BAG domain of Bag-1 (turquoise) and UbL domain of Bag-1 (purple) with the Rpn1 protein (brown). **e**—**j**. Close up views for interaction surfaces.

We showed that the BAG domain residues that are involved in the interaction with the ATPase domain of Hsp70 are concentrated on two alpha helices (α2 and α3) (Fig 4A), in accordance with the previous NMR and X-ray crystallography studies [44,45]. Structural alignment of the BAG domain from the crystal structure of ATP bound Hsc70–Bag-1 complex (PDB ID: 3FZF) and the BAG domain from the model of nucleotide free Hsp70–Bag-1 complex yielded an RMSD value of 2.736 Å and shared the residues on the interface more than 95%. This finding indicated that the Bag-1 and Hsp70/Hsc70 interaction was highly similar in ATP bound and unbound conditions.

Bag-1–VCP/p97 interaction model predicted that D1 domain of VCP/p97 interacts with BAG domain through two binding sites (Fig 4B). Hydrogen bonding between Q212 (BAG) and T688 (VCP/p97), and between T209 (BAG) and Q252 (VCP/p97) constitute the first interaction site. In the second interaction site, positively charged R161, R162 and K164 residues on BAG domain form hydrogen bonds and electrostatic interactions with the polar residues N401, E402, and Q473, respectively, on VCP/p97. Additionally, interactions of BAG domain with ER-resident proteins PDIA3, BiP (HSPA5), calnexin and calreticulin were estimated to be energetically favorable (S2 Table). However, these results are not biologically relevant as Bag-1 is not found in the ER lumen.

The UbL domain residues of Bag-1 that interact with UBA2 and UbL domains of Rad23B are mapped on the last three β-sheets that lie on the same face of the molecule (Fig 4C). Hydrophobic interactions are highly prevalent in these interactions. The hydrophobic stretch ^100^VMLIG^104^ of Bag-1 is predicted to interact with ^69^FVVVMV^74^ residues on UbL domain and ^9^RLKAL^13^ on UBA2 domain of Rad23B, respectively. The positively charged residue R9 on UBA2 domain makes a salt-bridge with E31 on the same domain, while interacting with the carbonyl oxygen of L102 on Bag-1’s UbL domain through hydrogen bonding (Fig 4C).

Bag-1 is predicted to bind Rpn1 (PSMD2; 26S proteasome non-ATPase regulatory subunit 2) both through its UbL and BAG domains (Fig 4D), suggesting that the well-established Bag-1–proteasome interaction might also involve the BAG domain in addition to the UbL domain. The interaction site on the BAG domain involves hydrogen bonding between R162 (BAG) and E157 (Rpn1). Moreover, E138 on BAG domain forms multiple salt bridges with R137, K141 and H161, and a backbone hydrogen bond with L162 on Rpn1. UbL–Rpn1 interaction is maintained by electrostatic interactions between K83 on UbL domain and E544 and E547 on Rpn1. Hydrogen bonds between K74 on UbL domain and S536 and T535 on Rpn1 further strengthen the interaction.

### Analysis of Bag-1’s interactions using mutations

Next, we analyzed the effects of mutations on the predicted interfaces between Bag-1 and its interaction partners using *in silico* tools and *in vitro* assays. Energetically important residues (i.e., hotspots) for protein-protein interactions were identified by Hotregion database (S7A and S7B Fig) [46]. We selected four residues predicted to be important for interactions with several components of the PQC systems. The BAG domain residues R161 and R193 were specifically selected for their involvement in Hsp70 binding, R161 and R162 for involvement in potential VCP/p97 binding, and the UbL domain residue I103 for involvement in potential Rad23B binding. Charge/polarity reversal mutations were designed for these residues, and their effects on the binding to specific interaction partners were predicted by using FoldX software [41,42]. The R161 R162E, R193D, and I103K mutations showed highly destabilizing effects on the interactions between Bag-1 and Rpn1, VCP/p97, Hsp70 and Rad23B, respectively (S7C Fig).

**Fig 5:**
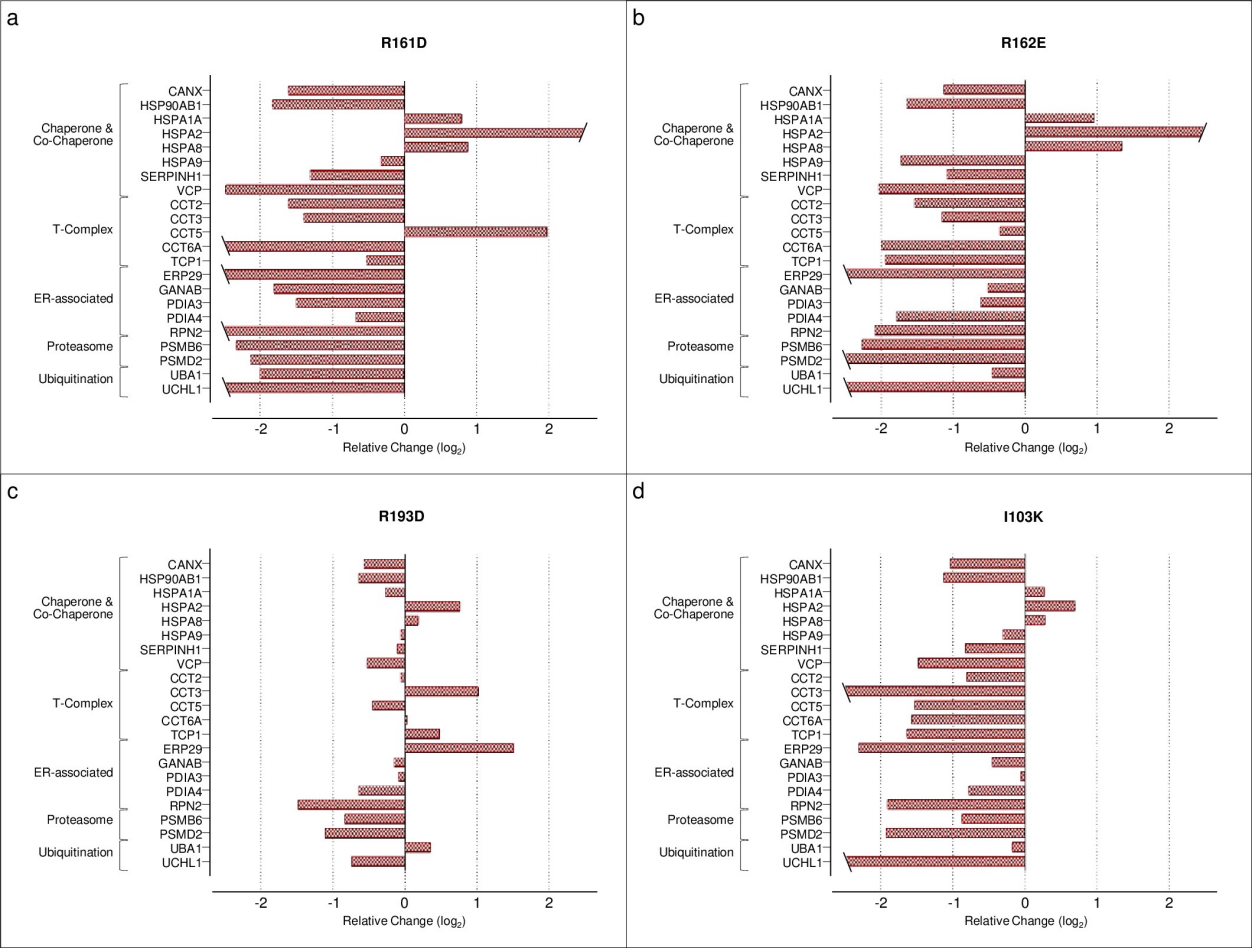
BAG and UbL mutants disrupt interaction of Bag-1 with protein quality control network. Protein abundances in the interactomes of TAP–Bag-1S mutants are compared to that of wild-type TAP–Bag-1S. Fold changes are depicted in log_2_ base for **a**. R161D, **b**. R162E, **c**. R193D, and **d**. I103K mutants.

To test these models experimentally, we generated mutations in TAP–Bag-1S by site-directed mutagenesis. Overexpression of these mutants in MCF-7 cells did not affect cell viability compared to wild-type Bag-1S (S8 Fig). We performed interactome analyses of Bag-1S mutants to quantify the relative changes in protein abundances between wild-type and mutant interactomes (S5 Table). R161D, R162E and I103K substitutions resulted in dramatic decreases in the abundances of VCP/p97, ER chaperones, and proteasome-ubiquitination components (Fig 5A–5C). In contrast, the abundance of many Hsp70 family chaperones in these mutant interactomes increased, possibly due to their chaperoning activity on the mutant proteins. However, unlike the other three mutations, R193D substitution resulted in a slight decrease in Hsp70 (HSPA1A) binding, as predicted by FoldX, and the abundance of other proteins were not as affected as in the case of other mutations (Fig 5D). Overall, the inverse correlation between the abundance of Hsp70 family chaperones and other Bag-1 interactors suggested that association of Bag-1 with the PQC elements might not be dependent on its interaction with Hsp70 chaperones.

### Mutations in Bag-1 affect proteasomal activity

**Fig 6:**
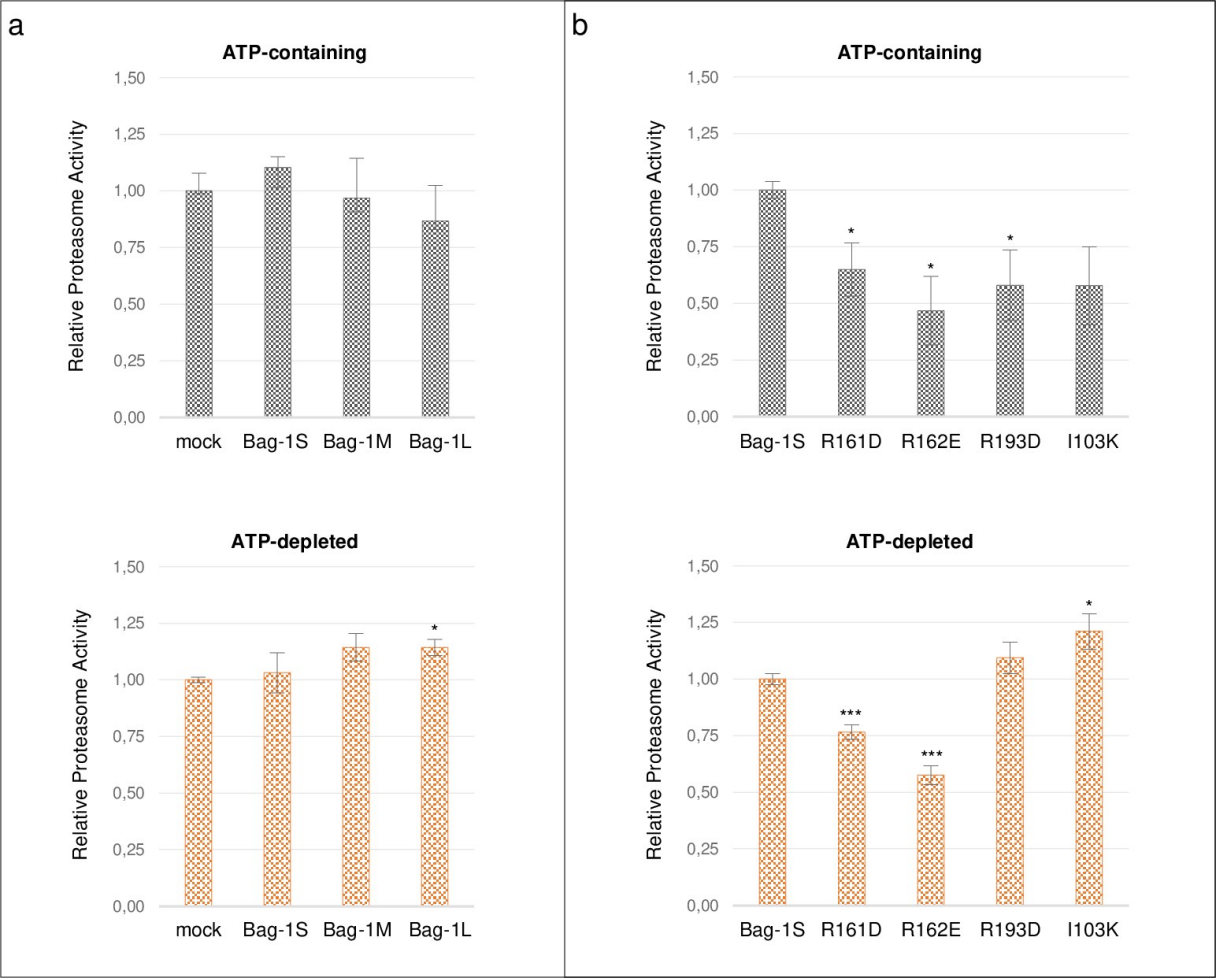
Mutations on BAG and UbL domains affect proteasomal activity. Proteasome activity was measured by the fluorescence of released MCA in protein extracts from MCF-7 cells after transfection with **a**. Bag-1 isoforms and mock vector, and **b**. Bag-1S mutants and wild-type Bag-1S. Proteasome activity was measured under ATP-containing (grey) and ATP-depleted (orange) conditions. Significant differences were determined by t-test.

Given the established interaction of Bag-1 with the proteasomal system, we addressed the effects of Bag-1 isoforms and Bag-1 mutants on proteasome activity using an *in vitro* assay. Proteasomal degradation of a fluorogenic peptide substrate was measured in MCF-7 cells transfected with Bag-1 isoforms and mock vector, or with mutant and wild-type Bag-1S. Bag-1S, M or L isoforms did not significantly alter the proteasome activity compared to mock in both ATP-depleted and ATP-containing conditions (Fig 6A). Bag-1S mutants R161D and R162E significantly decreased proteasome activity compared to wild-type Bag-1S in both conditions, whereas R193D mutant significantly decreased proteasomal degradation only in ATP-containing condition without causing a significant change in ATP-depleted condition (Fig 6B). Lastly, I103K mutant significantly increased proteasomal degradation in ATP-depleted condition. Overall, mutations on BAG domain of Bag-1 resulted in impaired proteasomal activity.

### Bag-1 affects degradation of ERAD substrate CD147

**Fig 7:**
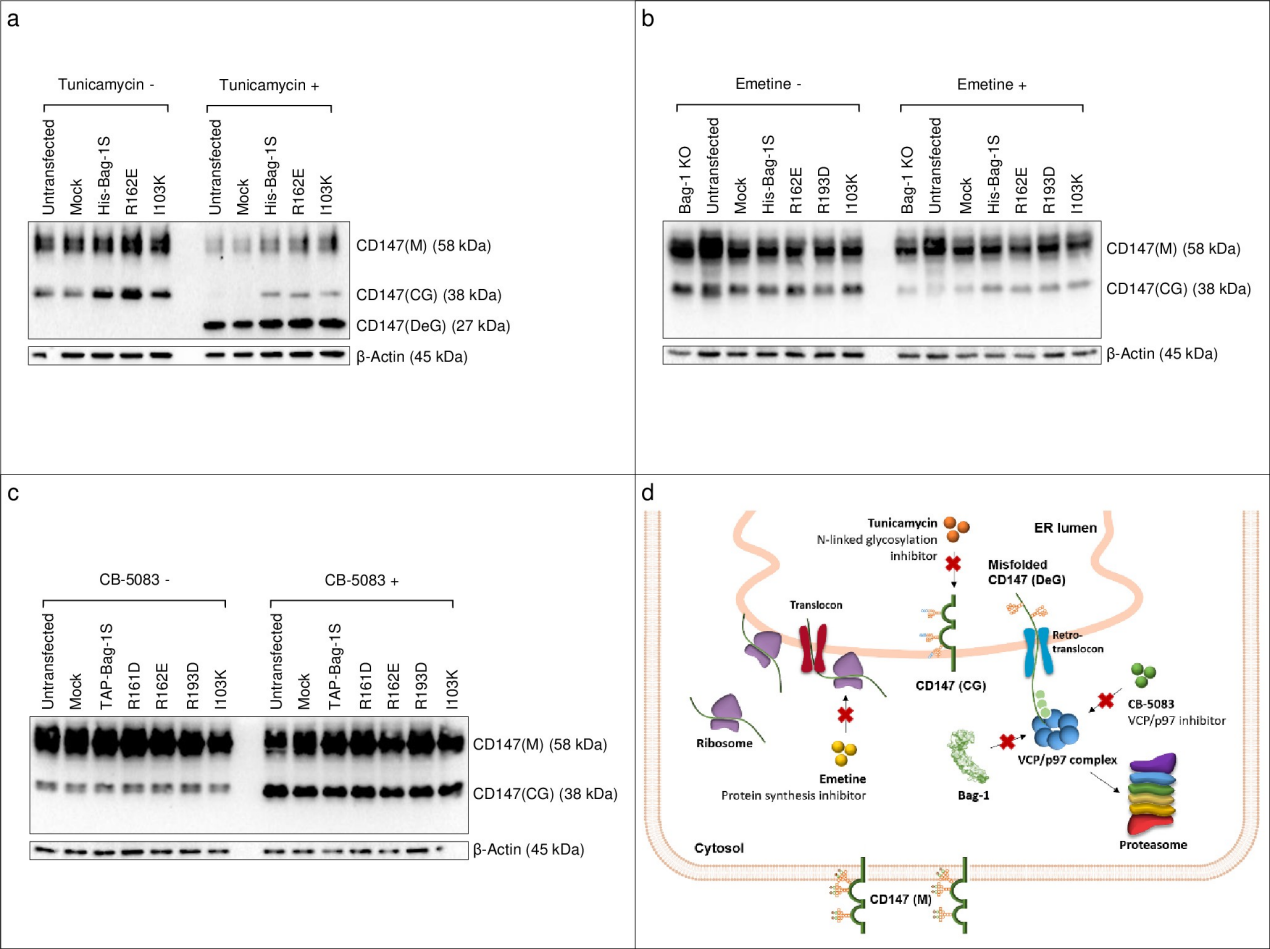
Bag-1 downregulates VCP/p97-dependent ER-associated degradation of CD147. Bag-1 KO MCF-7 cells were transfected with mock vector, wild-type Bag-1S and mutant Bag-1S, and treated with **a**. glycosylation inhibitor tunicamycin, **b**. translation inhibitor emetine, **c**. VCP/p97 inhibitor CB-5083. Immunoblots of CD147 (M: Mature, CG: Core glycosylated, DeG: Deglycosylated) are shown in **a-c**. **d**. model for downregulation of CD147 degradation by Bag-1.

The association of Bag-1 with VCP/p97 and the presence of ER resident proteins in Bag-1 interactomes led us to speculate that Bag-1 might play a role in ERAD. To test whether Bag-1 regulates ERAD activity, we selected a known ERAD and VCP/p97 substrate, the transmembrane protein named CD147, and investigated the effect of overexpression of wild-type and mutant Bag-1 isoforms on its degradation in Bag-1 KO MCF-7 cells. CD147 can be found in mature (M), core glycosylated (CG) and deglycosylated (DeG) forms. CD147(M) is found on the plasma membrane; CD147(CG) is found in the ER and is a constitutive substrate for VCP/p97-dependent degradation; and CD147(DeG) appears before proteasomal degradation [47,48]. We observed that treatment with tunicamycin, which inhibits glycosylation and triggers ER stress, caused disappearance of CD147(CG) and appearance of CD147(DeG) in wild-type and mock-overexpressing KO cells (Fig 7A). Strikingly, however, in Bag1S overexpressing cells, CD147(CG) was preserved at considerable levels. This suggested that Bag-1S downregulated the degradation of CD147(CG) upon ER stress. Bag-1S mutants resulted in preservation of CD147(CG) form at similar levels with wild-type Bag-1S, suggesting that mutants might not be fully effective in the negative regulation of ERAD. Treatment of wild-type and mock overexpressing KO cells with the translation inhibitor emetine caused significant decreases in CD147(M) and CD147(CG) forms (Fig 7B). However, cells overexpressing wild-type or mutant Bag-1S showed a decrease in CD147(CG) to a lesser extent. CD147(CG) was absent in immunoblots of KO cells treated with both tunicamycin and emetine (S9 Fig) indicating that tunicamycin caused CD147(DeG) accumulation in two ways, i.e., by blocking the formation of CD147(CG) after translation and by triggering deglycosylation of available CD147(CG) under ER stress. Lastly, treatment with VCP/p97 inhibitor CB-5083 resulted in a prominent accumulation of CD147(CG) (Fig 7C). However, there were no difference in CD147(CG) levels among wild-type, mock-overexpressing and Bag-1S-overexpressing cells upon treatment with CB-5083. These results indicated that Bag-1S overexpression in KO cells partially protected CD147(CG) from degradation, having a similar negative effect on ERAD as the VCP/p97 inhibitor. The protective effect of Bag-1S was not visible when VCP/p97 activity was fully blocked by a chemical agent. Altogether, we propose that Bag-1S negatively regulates VCP activity and ERAD (Fig 7D).

## Discussion

This study is a systematic investigation of isoform-specific interaction partners of human Bag-1 protein using tandem MS proteomics. Interactome analysis revealed largely overlapping pathways for Bag-1 isoforms. The interactomes of Bag-1 isoforms were enriched with proteins involved in protein homeostasis, including many novel interaction partners. Chaperones, ER-resident proteins, ubiquitination elements and proteasome subunits co-existed with Bag-1 isoforms in complexes. These novel interactions suggested that the role of Bag-1 in protein homeostasis might be broader than previously anticipated.

The interactome analysis confirmed well-known interactions of Bag-1 with Hsp70/Hsc70 [14] and proteasome subunits [49], and revealed novel candidate interacting partners. Many of these proteins may not be direct interaction partners of Bag-1, but may rather be part of complexes formed by Bag-1-binding chaperones, as suggested previously [50]. These putative interactions warrant further validation. In this study, we particularly studied the interactions of Bag-1 with VCP/p97, Rad23B, and Rpn1 using *in silico* tools and/or *in vitro* assays.

We predicted that Bag-1 interacts with VCP/p97 through its BAG domain, and mutants in BAG domain partially disrupt this interaction. VCP/p97 is an AAA+ ATPase, which segregates ubiquitinated substrates from their binding proteins and mediates the retrotranslocation of misfolded proteins from the ER into the cytosol for proteasomal degradation [51,52]. We speculated that Bag-1 might be associated with ERAD machinery through VCP/p97 binding. To test whether Bag-1 regulates degradation of ER proteins, we followed CD147 glycosylation pattern in control and Bag-1S overexpressing Bag-1 KO cells upon treatment with different inhibitors. We observed that CD147(CG) was deglycosylated, and CD147(DeG) accumulated under tunicamycin-induced ER stress conditions. The conversion of CD147(CG) to CD147(DeG) was complete in wild-type and mock-expressing cells. In contrast, in the presence of wild-type or mutant Bag-1S, CD147(CG) was partially preserved, suggesting that Bag-1 downregulates VCP/p97-dependent ERAD. Further research should investigate the exact mechanism of Bag-1’s function in ERAD. Three important questions remain to be answered: 1) Does Bag-1’s effect on ERAD rely on VCP/p97 binding? 2) Degradation of which proteins does Bag-1 affect? and 3) Does Bag-1 have only downregulatory effect on ERAD?

Many ER-specific chaperones including PDI, ribophorin, calreticulin and calnexin were identified in Bag-1 isoform interactomes. These chaperones are responsible for the recognition, refolding or tagging of misfolded proteins in the ER [53]. We predicted potential direct interactions using *in silico* tools. However, these proteins are physically found in different compartments of the cell, and hence their direct interactions with Bag-1 seem to be not plausible. Their presence in Bag-1 interactome has two possible explanations. First, interaction of Bag-1 and VCP/p97 carried these proteins despite several purification steps. The cytosolic VCP/p97 protein is recruited to ER membranes by interacting with the VCP-interacting membrane protein (VIMP), which comes into contact with ER lumen chaperones during ERAD [54]. Second, interaction of Bag-1 with ER-resident proteins may occur after cell lysis. Bag-1 was previously found to interact with the ER-resident BiP and GADD34 proteins *in vitro* [55,56]. Given that Bag-1 is not in the ER lumen, direct binding of Bag-1 with ER lumen proteins is possible only after cell lysis.

Bag-1 regulates chaperone activity either positively or negatively in a concentration and isoform-dependent manner, assisting in either protein folding or protein degradation [57,58]. In accordance with its role in protein degradation, Bag-1 promotes the tethering of Hsp70/Hsc70 chaperones to the proteasome by simultaneously binding them through its BAG and UbL domains, respectively [54]. Hence, Bag-1 may function in the funneling of chaperone clients into the proteasome for degradation. Here, we predicted that Bag-1 UbL binds UBA2 and UbL domains of Rad23B. Even though UbL-UbL interaction has not been reported in the literature, intermolecular and intramolecular UbL-UBA interactions have been extensively documented [59]. Rad23B is a polyubiquitin chain receptor, which delivers ubiquitinated proteins to 26S proteasome [60]. Whether Bag-1 UbL competes with Rad23B UbL for proteasome binding, or with ubiquitinated proteins for Rad23B UBA2 binding is an interesting question and remains to be answered.

An important aspect of this interactome analysis was the inclusion of a negative control, *i*.*e*., TAP-only mock vector. Relative quantitation of proteins in Bag-1 isoform immunoprecipitates in comparison to this control ensured that the identified binding partners were Bag-1-dependent. We used multiple peptide search engines and consensus filtering to improve annotation of peptide fragments and increase the depth of the interactome analysis. Despite this, some of the known binding partners of Bag-1, including Bcl-2, CHIP, Raf and Akt, were not detected in interactome analysis. These proteins most probably engage in transient interactions with Bag-1 that are not strong enough to persist during affinity purification. It is unlikely that the binding of these proteins to Bag-1 was hindered by the N-terminal CBP peptide, which remained covalently bound after TEV cleavage, because they were also not detected in the interactome of the C-terminal TAP-tagged Bag-1. Another shortcoming of MS-based interactomics is that detection of interactors was affected by the expression levels of proteins. Despite these drawbacks, the implemented method provided a robust profile of Bag-1 interacting partners, especially for stable interactions.

Small molecule inhibitors that disrupted Bag-1’s interactions at the C-terminal end were previously found to inhibit the growth of breast cancer cells [61]. Here, we showed that amino acid substitution mutations on these domains disrupted the association of Bag-1 with PQC elements. We also found that Bag-1S mutations, especially R161D and R162E in the BAG domain, resulted in substantially decreased ATP dependent degradation capacity of proteasomes. Interestingly, according to our *in silico* analyses, R161 and R162 were predicted to be important for binding to Rpn1, the regulatory subunit of the 26S proteasome (S2 Table). The UbL domain mutation I103K significantly increased ATP-depleted proteasomal activity, but insignificantly decreased ATP-containing proteasomal activity. Therefore, both BAG and UbL domains might be involved in interaction of Bag-1 with the proteasome. Notably, Hsp70 family proteins significantly increased while other PQC elements decreased in the interactomes of R161D, R162E and I103K mutants compared to wild-type Bag-1S interactome, suggesting a lack of proper folding in the mutants. Therefore, it should also be noted that the inhibition of proteasomal degradation by Bag-1S mutants might also result from mutant proteins plugging the proteasome. Another BAG family protein Bag-6, which also contains both BAG and UbL domains, was shown to regulate the assembling of the core and regulatory particles of 26S proteasome [62]. The exact contribution of Bag-1 to proteasome activity is still unknown. Whether Bag-1 assumes a similar function in proteasome assembly must be further investigated.

Overall, in this study we characterized the isoform-specific interactomes of Bag-1 and identified novel interacting partners. We propose that Bag-1 plays a role in ERAD, in addition to cytosolic degradation, and modulates proteasome activity through these interactions. The interaction surfaces of Bag-1 with key elements of PQC systems were modelled, and critical residues were analyzed using mutations. The structures of these complexes must be further studied with high-resolution techniques to guide rational drug design studies for targeting PQC pathways in the treatment of cancer.

## Supporting information

S1 Fig Overexpression of Bag-1 isoforms enhances cell proliferation. (DOCX)

S2 Fig Uncropped western blot scans displayed in Fig 3A. (DOCX)

S3 Fig Western blot scans of TAP-purification controls from MCF-7 and MCF-12A untransfected cells. (DOCX)

S4 Fig Western blot scans of Bag-1 IP from MCF-7 and MCF-12A untransfected cells. (DOCX)

S5 Fig ICC experiments for wild-type MCF-7 cells and Bag-1 KO MCF-7 cells. (DOCX)

S6 Fig Uncropped western blot scans of Co-IP from tumor and normal tissue samples. (DOCX)

S7 Fig Predicted hotspot residues in BAG and UbL domains of Bag-1. (DOCX)

S8 Fig Cell proliferation assay for Bag-1 mutants in MCF-7 cells. (DOCX)

S9 Fig Effect of double treatment with tunicamycin and emetine on CD147. (DOCX)

S1 Table Computational predictions for mutant BAG and UbL domains. (XLSX)

S2 Table Computational predictions for BAG and UbL domains of Bag-1. (XLSX)

S3 Table List of proteins identified in Bag-1 isoform interactomes relative to mock interactome. (XLSX)

S4 Table List of proteins identified in the BN-PAGE gel. (XLSX)

S5 Table List of proteins identified in mutant Bag-1 interactomes relative to Bag-1S interactome. (XLSX)
