# Supplementary material for: Interactome analysis of Bag-1 isoforms reveals novel interaction partners in endoplasmic reticulum-associated degradation
Source: PLoS One. 2021 Aug 24;16(8):e0256640. doi: 10.1371/journal.pone.0256640 (PMC8384158; doi:10.1371/journal.pone.0256640)
Supplement: S1 Fig — (DOCX) [file pone.0256640.s001.docx]

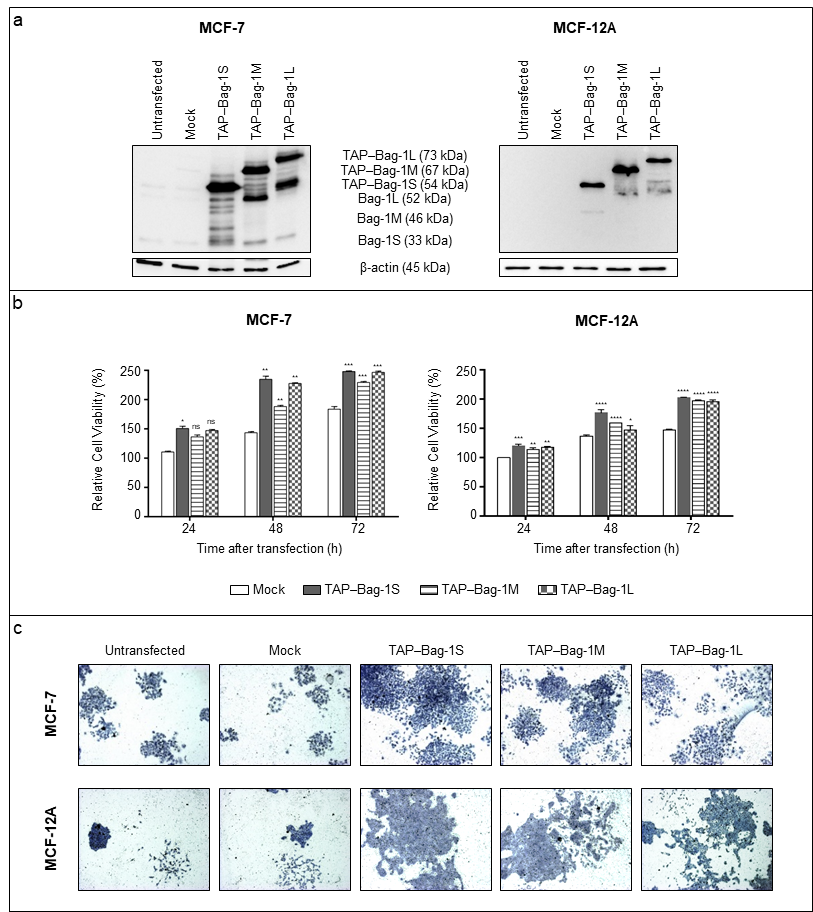


**Figure S1. Overexpression of Bag-1 isoforms enhances cell proliferation. a**. Immunoblotting of protein lysates from MCF-7 and MCF-12A cells transfected with the mock vector and N-terminal TAP-tagged Bag-1 isoforms, as well as from untransfected cells. Bag-1 isoforms were blotted with anti-Bag-1 antibody. Ectopically expressed Bag-1S, M and L isoforms were detected at 54, 67 and 73 kDa respectively, due to additional weight of TAP tag (21 kDa). **b**. MTT assay and **c**. Colony formation assay for evaluation of cell viability after transfection with TAP–Bag-1 isoforms. Results for MTT assay are shown relative to mock vector. Significant differences were determined by t-test.
