# Supplementary material for: Interactome analysis of Bag-1 isoforms reveals novel interaction partners in endoplasmic reticulum-associated degradation
Source: PLoS One. 2021 Aug 24;16(8):e0256640. doi: 10.1371/journal.pone.0256640 (PMC8384158; doi:10.1371/journal.pone.0256640)
Supplement: S2 Fig — (DOCX) [file pone.0256640.s002.docx]

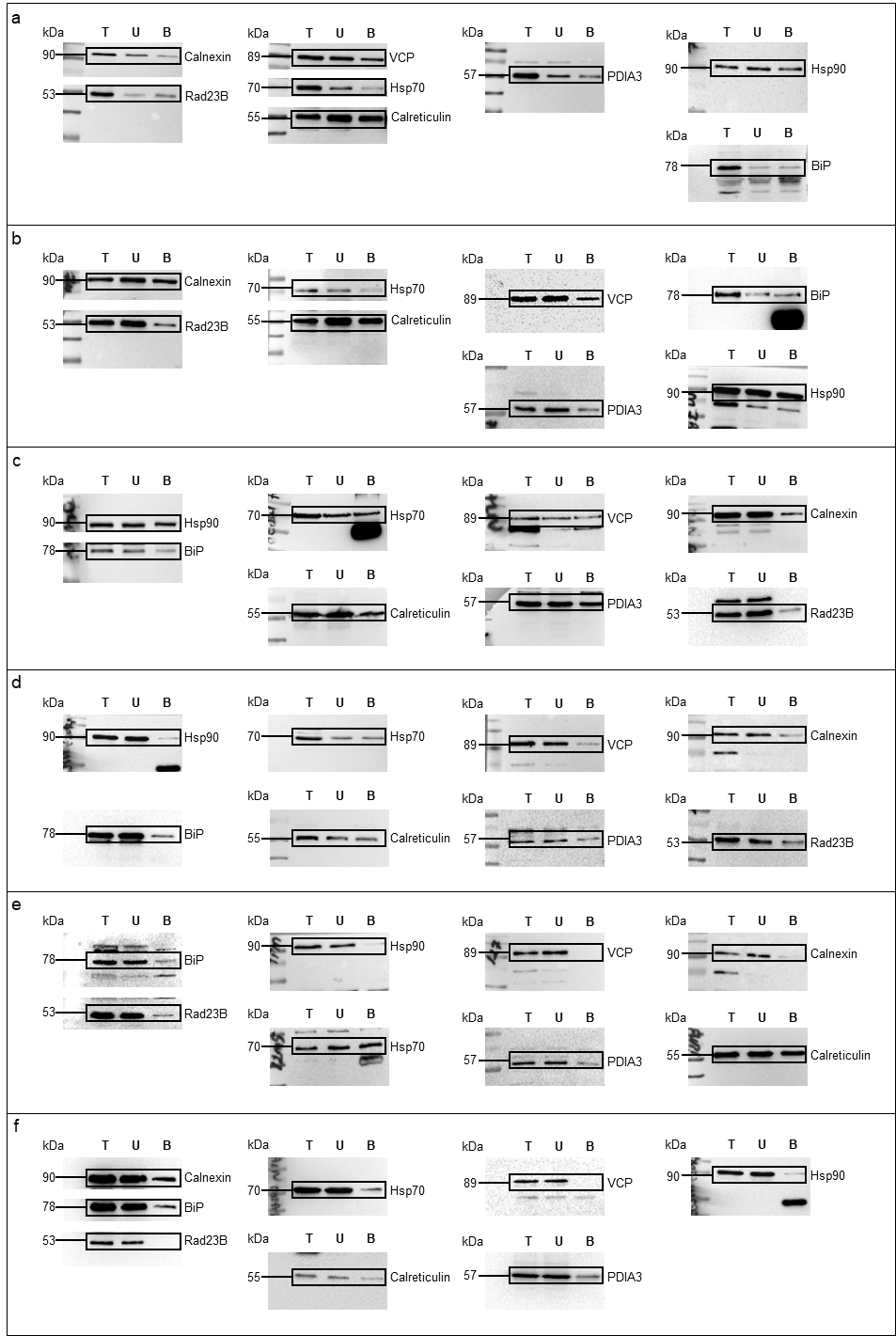


**Figure S2. Uncropped western blot scans displayed in Figure 3a.** TAP purification from MCF-7 cells transfected with **a**. TAP–Bag-1S, **b**. TAP–Bag-1M and **c**. TAP–Bag-1L vector, and MCF-12A cells transfected **d**. TAP–Bag-1S, **e**. TAP–Bag-1M and **f**. TAP–Bag-1L vector (T: total cell lysate, U: unbound, B: bound).
