# Supplementary material for: Interactome analysis of Bag-1 isoforms reveals novel interaction partners in endoplasmic reticulum-associated degradation
Source: PLoS One. 2021 Aug 24;16(8):e0256640. doi: 10.1371/journal.pone.0256640 (PMC8384158; doi:10.1371/journal.pone.0256640)
Supplement: S3 Fig — (DOCX) [file pone.0256640.s003.docx]

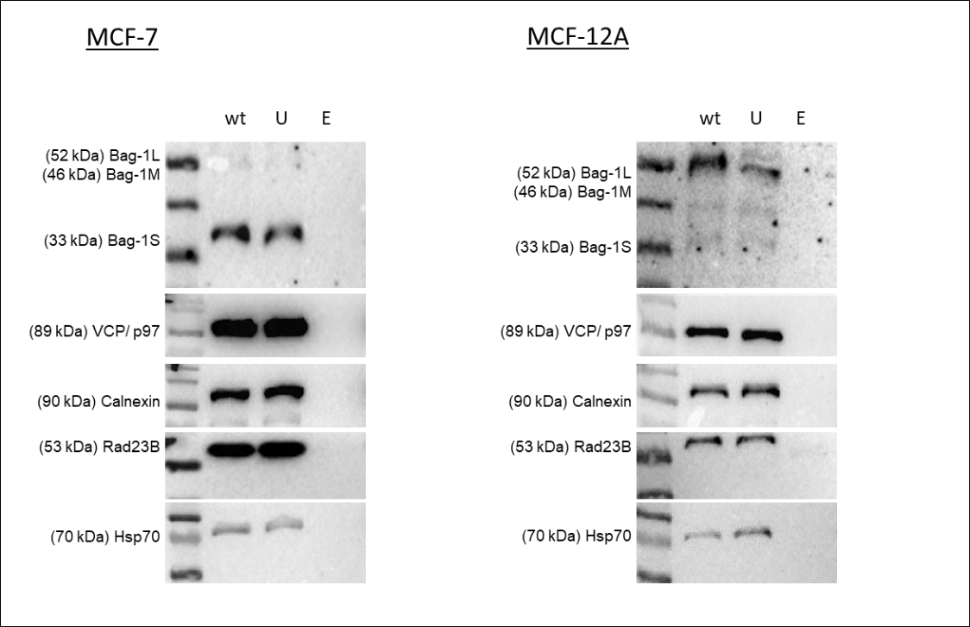


**Figure S3. Western blot scans of TAP-purification controls from MCF-7 and MCF-12A untransfected cells.** Immunoblots of TAP purification from untransfected MCF-7 and MCF-12A cells. Bag-1, VCP/p97, calnexin, Rad23B and Hsp70 was detected in the total protein lysate and unbound lane, whereas no association were detected in the elution for both cell lines (wt: wild type, U: unbound, E: elution).
