# Supplementary material for: Interactome analysis of Bag-1 isoforms reveals novel interaction partners in endoplasmic reticulum-associated degradation
Source: PLoS One. 2021 Aug 24;16(8):e0256640. doi: 10.1371/journal.pone.0256640 (PMC8384158; doi:10.1371/journal.pone.0256640)
Supplement: S4 Fig — (DOCX) [file pone.0256640.s004.docx]

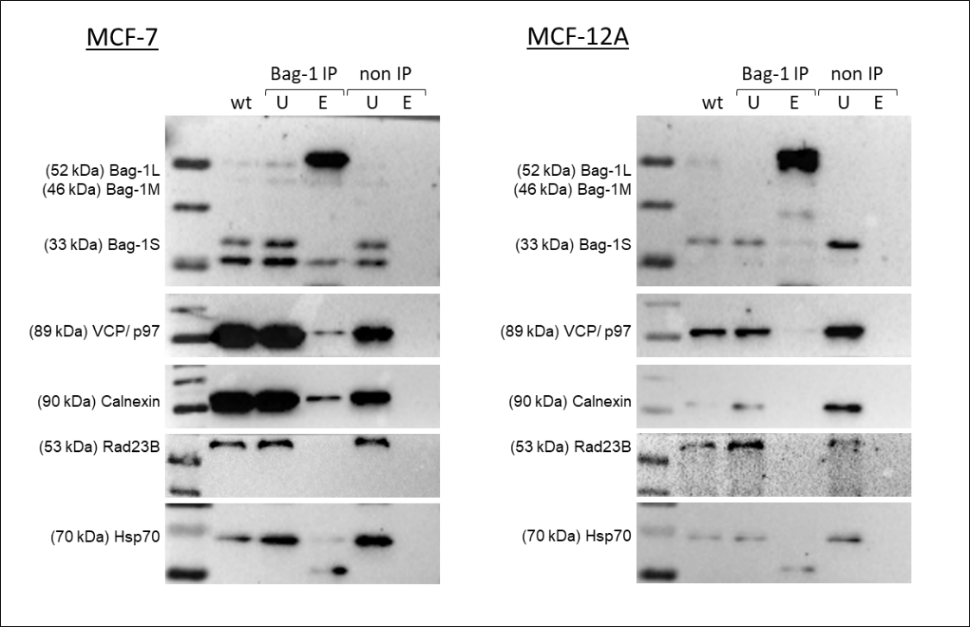


**Figure S4. Western blot scans of Bag-1 IP from MCF-7 and MCF-12A untransfected cells.** Immunoblots of co-IP from untransfected MCF-7 and MCF-12A cells. To eliminate the resin-related associations, 250 µg total protein lysate were incubated with antibody bound (Bag-1 IP) and unbound (non IP) Protein G beads. Immunoprecipitates were blotted for Bag-1, VCP, calnexin, Rad23B and Hsp70 (wt: wild type, U: unbound, E: elution).
