# Supplementary material for: Interactome analysis of Bag-1 isoforms reveals novel interaction partners in endoplasmic reticulum-associated degradation
Source: PLoS One. 2021 Aug 24;16(8):e0256640. doi: 10.1371/journal.pone.0256640 (PMC8384158; doi:10.1371/journal.pone.0256640)
Supplement: S5 Fig — (DOCX) [file pone.0256640.s005.docx]

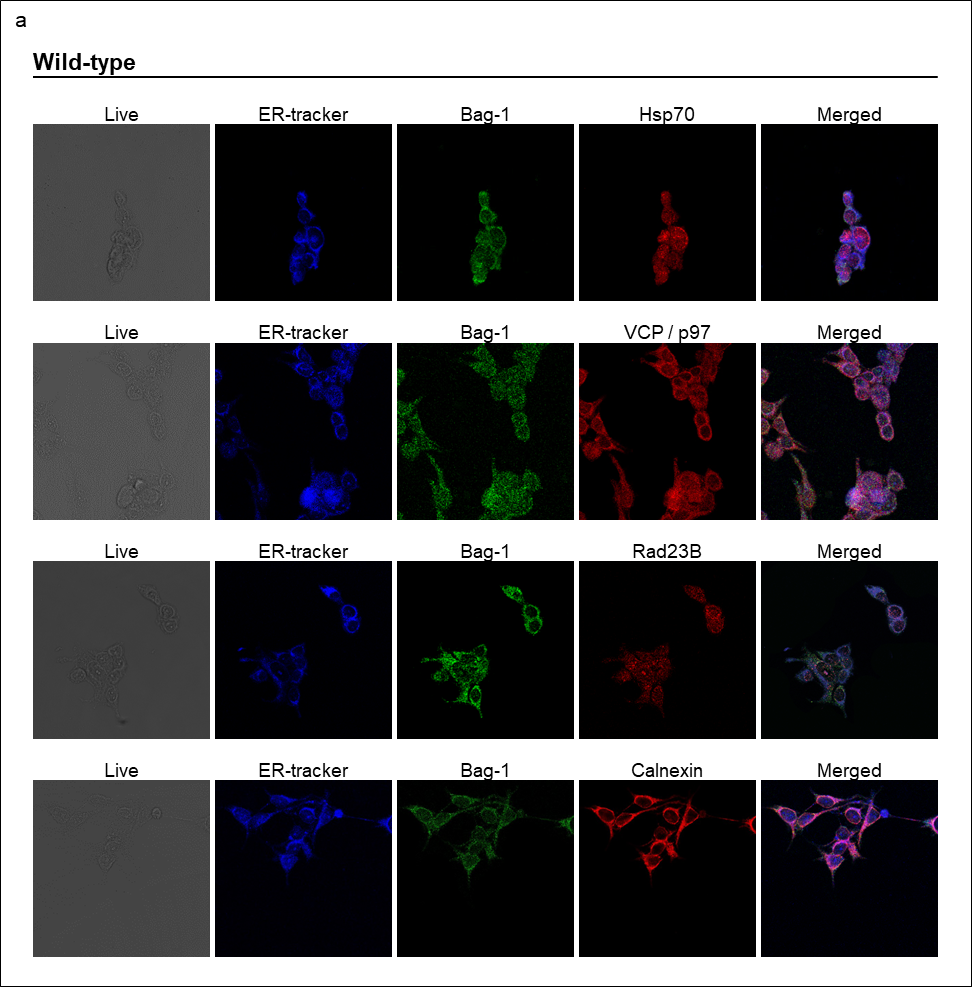


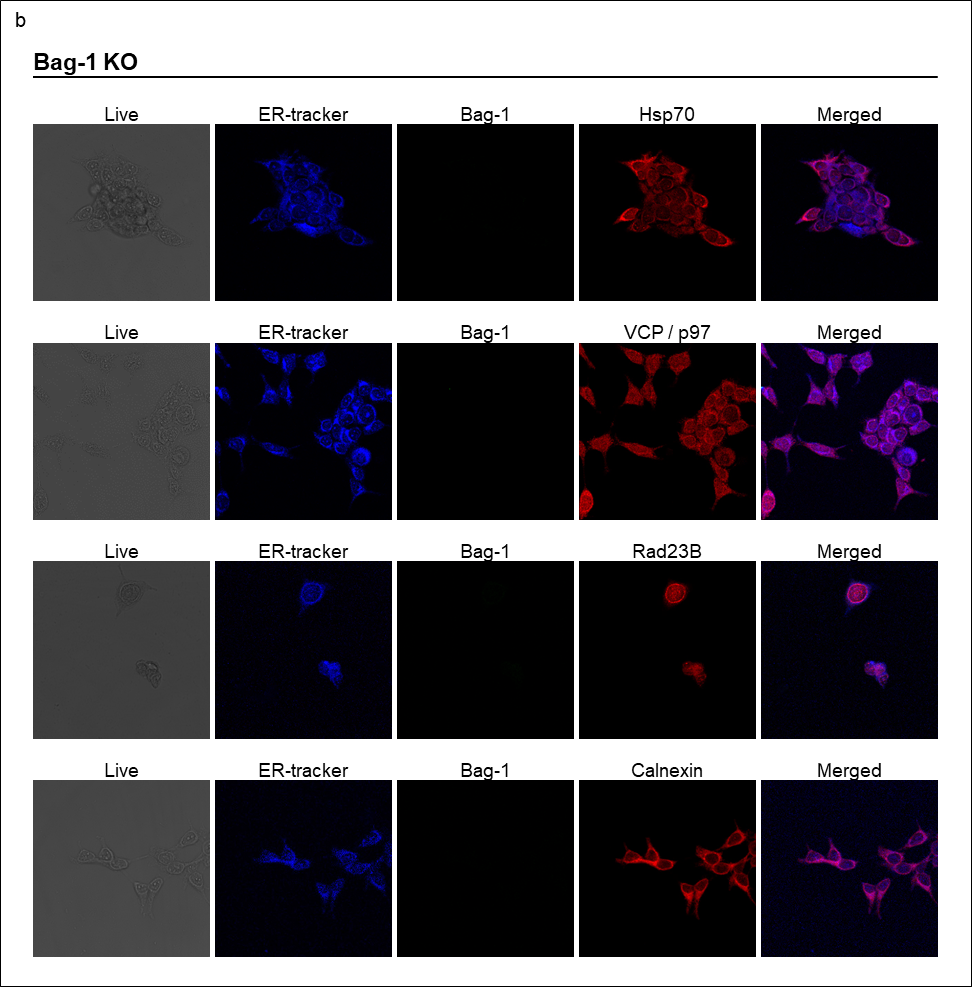


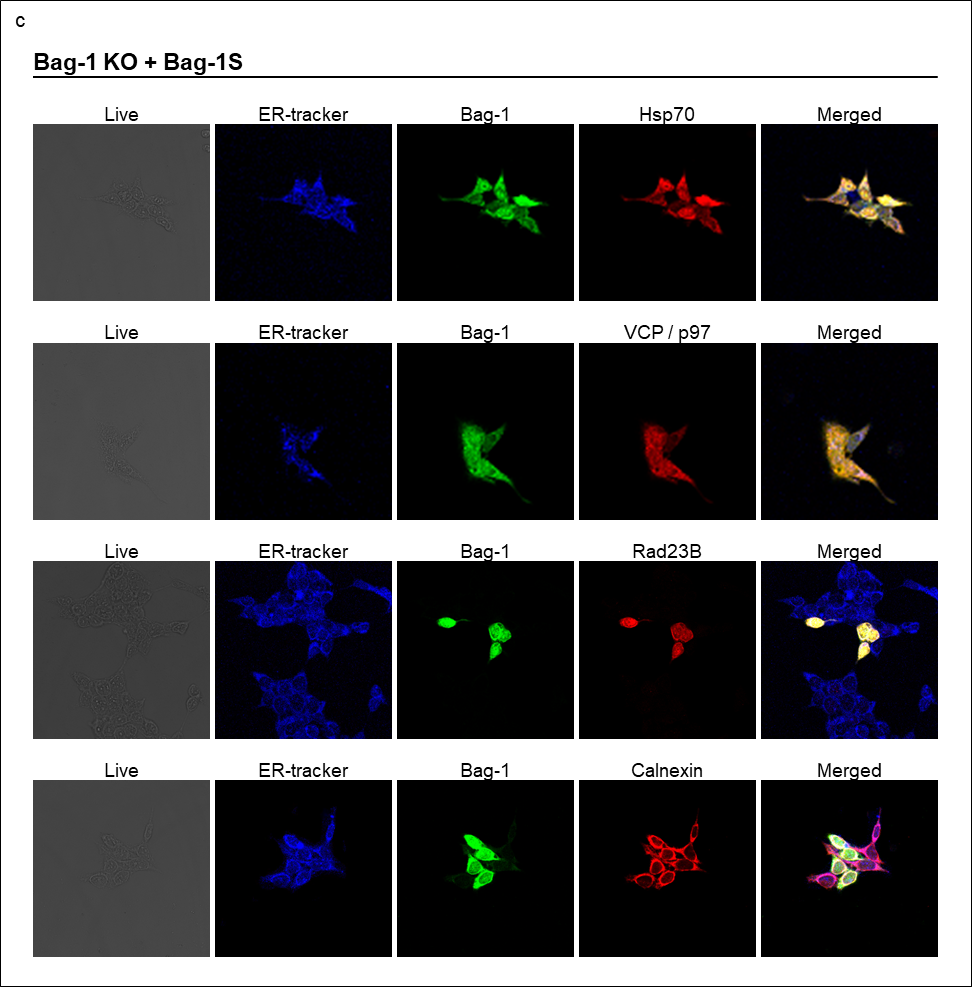


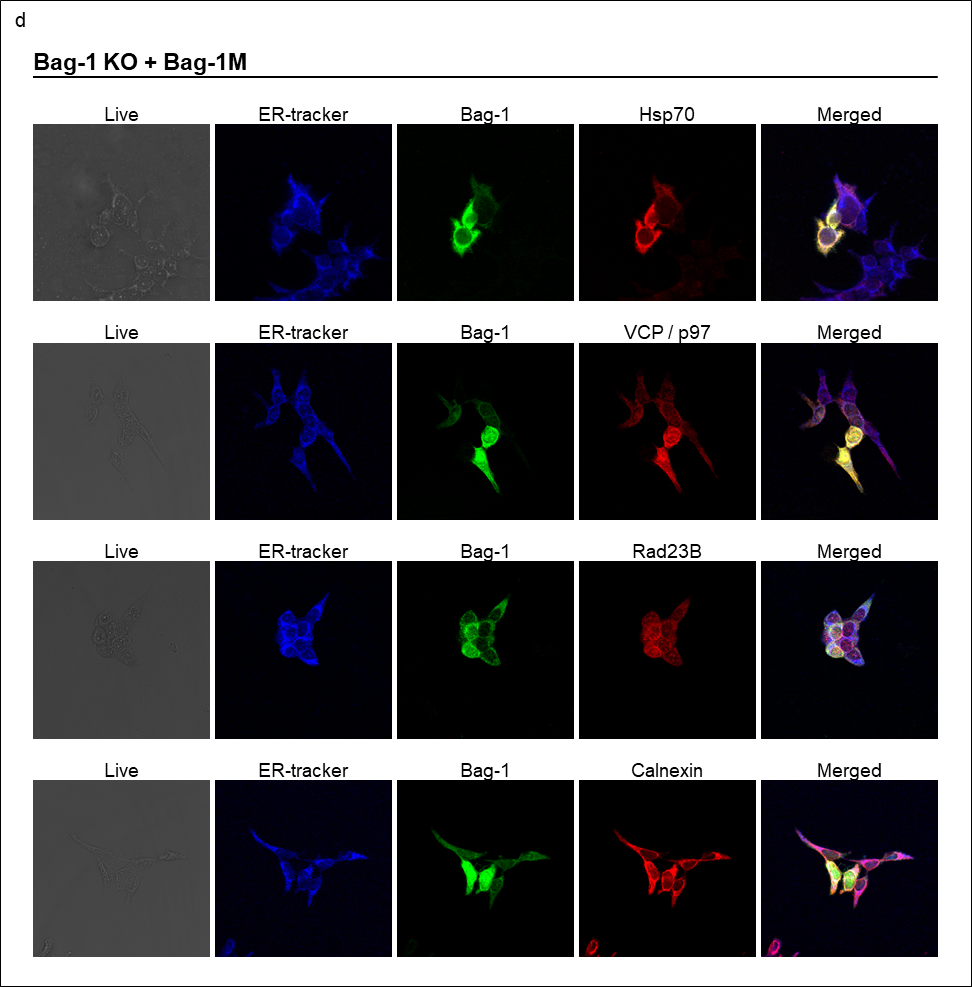


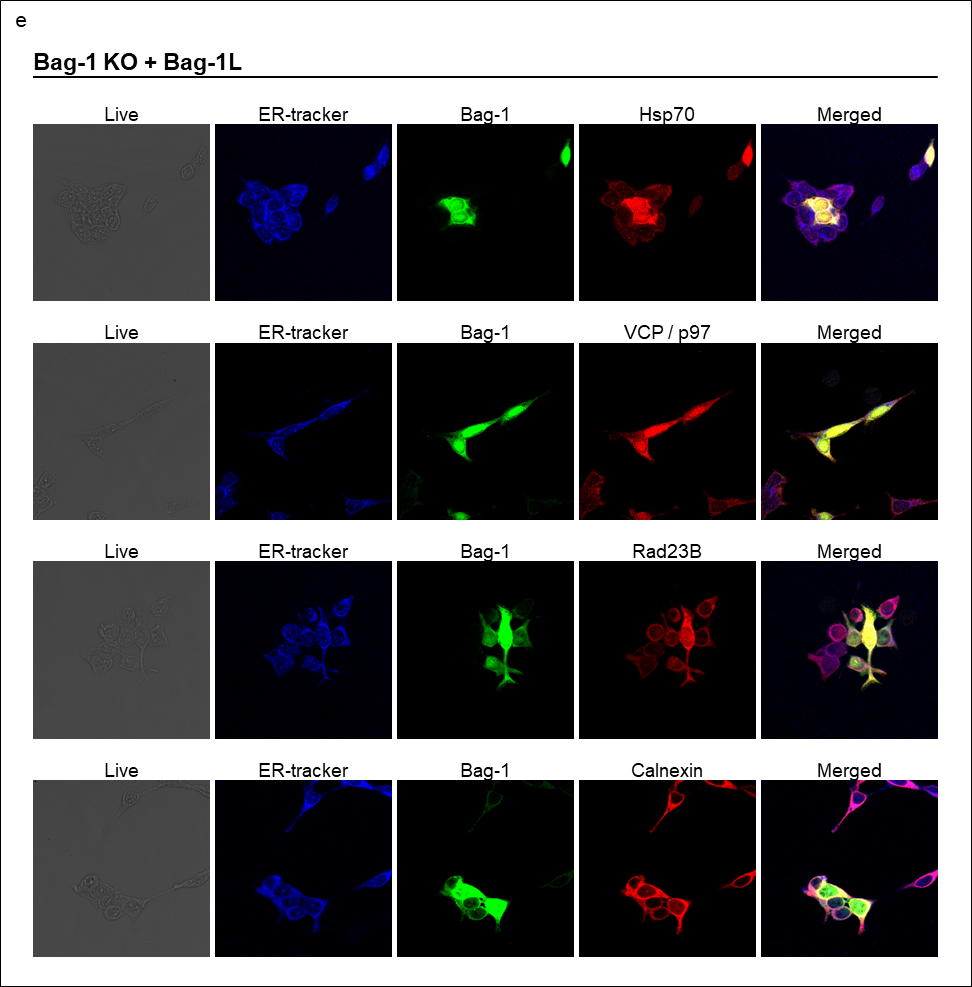


**Figure S5. ICC experiments for wild-type MCF-7 cells and Bag-1 KO MCF-7 cells. a**. Wild-type MCF-7 cells, **b**. Bag-1 KO MCF-7 cells, **c-e**. TAP-Bag-1 isoforms transfected Bag-1 KO MCF-7 cells were stained with Bag-1 antibody and VCP/p97, Rad23B or calnexin antibody. Hsp70 was used as a positive control.
