# Supplementary material for: Interactome analysis of Bag-1 isoforms reveals novel interaction partners in endoplasmic reticulum-associated degradation
Source: PLoS One. 2021 Aug 24;16(8):e0256640. doi: 10.1371/journal.pone.0256640 (PMC8384158; doi:10.1371/journal.pone.0256640)
Supplement: S6 Fig — (DOCX) [file pone.0256640.s006.docx]

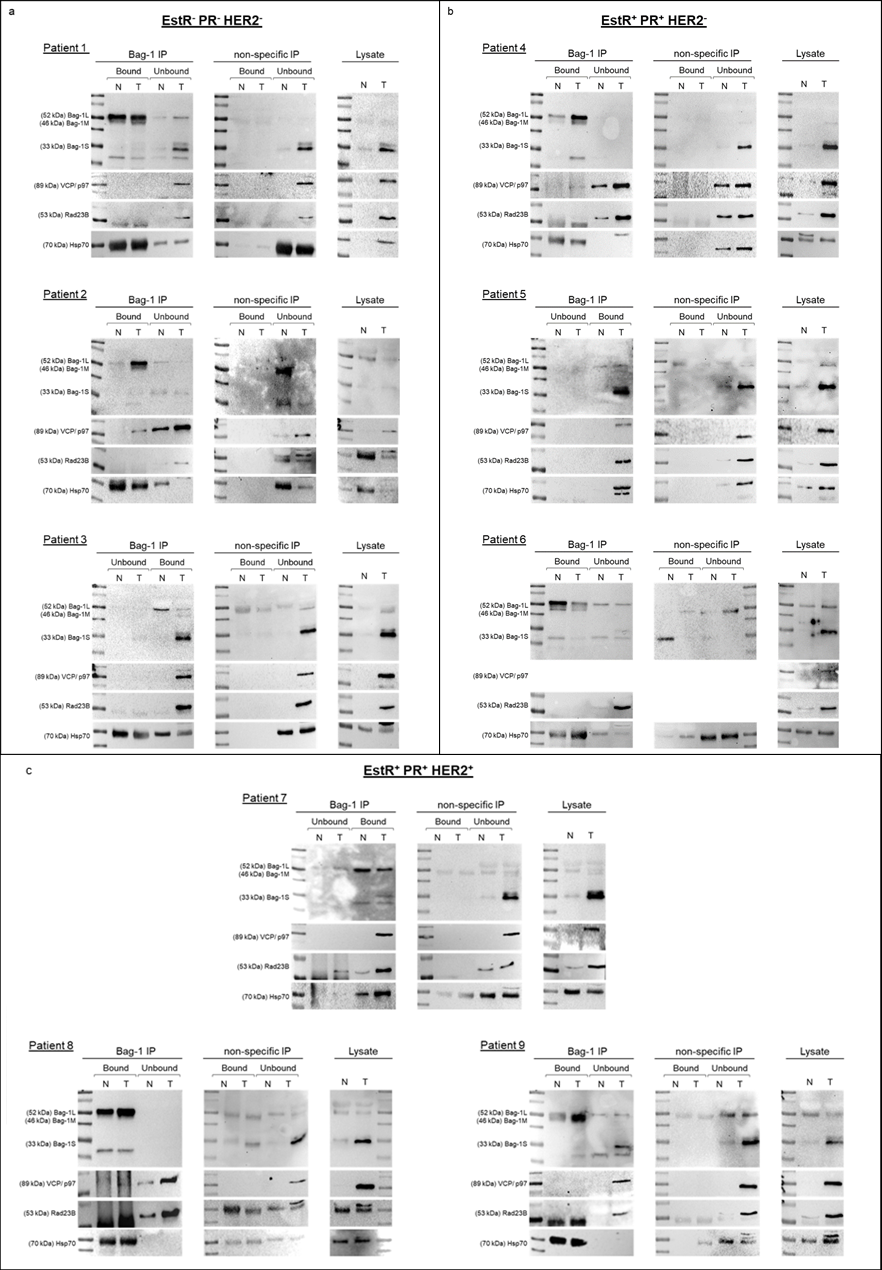


**Figure S6. Uncropped western blot scans of Co-IP from tumor and normal tissue samples.** Protein extracts were incubated with antibody bound (Bag-1 IP) and unbound (non-specific IP) Protein G beads. Immunoprecipitates were blotted for Bag-1, VCP, Rad23B and Hsp70. **a**. EstR- PR- HER2-, **b**. EstR+ PR+ HER2-, **c**. EstR+ PR+ HER2+ (N: normal tissue, T: tumor tissue).
