# Supplementary material for: Interactome analysis of Bag-1 isoforms reveals novel interaction partners in endoplasmic reticulum-associated degradation
Source: PLoS One. 2021 Aug 24;16(8):e0256640. doi: 10.1371/journal.pone.0256640 (PMC8384158; doi:10.1371/journal.pone.0256640)
Supplement: S7 Fig — (DOCX) [file pone.0256640.s007.docx]

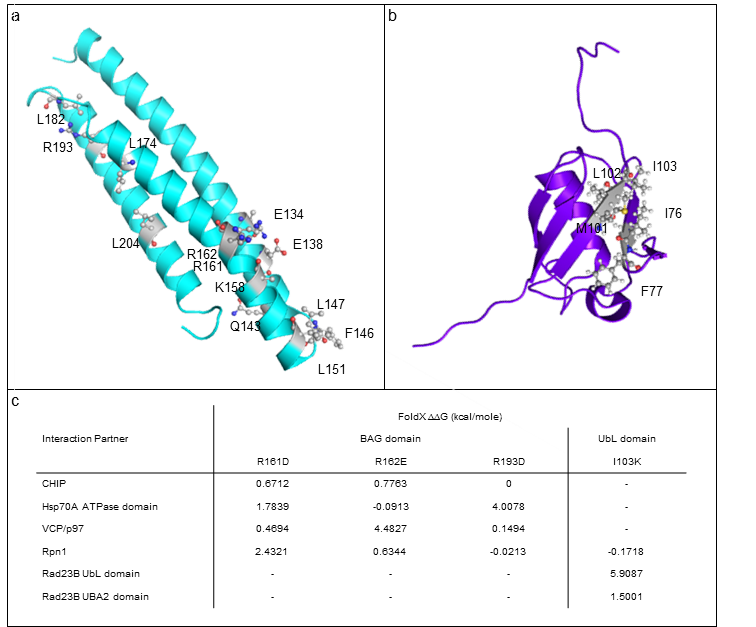


**Figure S7. Predicted hotspot residues in BAG and UbL domains of Bag-1.** Key residues in **a**. BAG domain and **b**. UbL domain are shown in balls and sticks. Important residues with more than three interactions are colored in gray. **c**. Gibbs energy changes for Bag-1 mutants’ interactions with the binding partners calculated by FoldX. Results are shown for R161D, R162E, R193D and I103K. Mutant categories on the basis of ∆∆G (kcal/mole) values are as following: neutral for -0.46 < ∆∆G ≤ 0.46; slightly destabilizing for 0.46 < ∆∆G ≤ 0.92; destabilizing for 0.92 < ∆∆G ≤ 1.84; highly destabilizing for ∆∆G > 1.84.
