# Supplementary material for: Interactome analysis of Bag-1 isoforms reveals novel interaction partners in endoplasmic reticulum-associated degradation
Source: PLoS One. 2021 Aug 24;16(8):e0256640. doi: 10.1371/journal.pone.0256640 (PMC8384158; doi:10.1371/journal.pone.0256640)
Supplement: S8 Fig — (DOCX) [file pone.0256640.s008.docx]

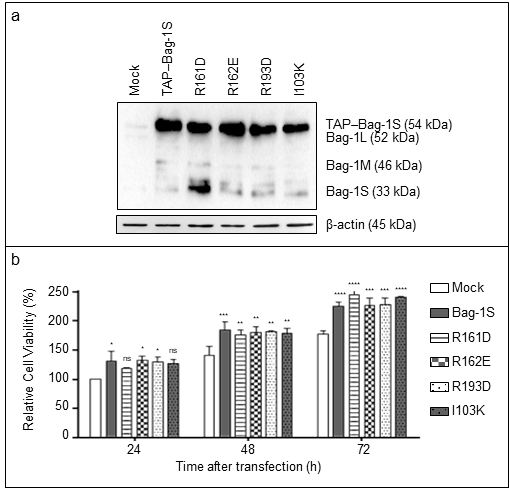


**Figure S8. Cell proliferation assay for Bag-1 mutants in MCF-7 cells. a**. Immunoblots of protein lysates from MCF-7 cells transfected with mock vector, TAP–tagged Bag-1S, and TAP–Bag-1S mutants R161D, R162E, R193D and I103K. Protein lysates were blotted by anti-Bag-1 antibody. **b**. MTT assay. Cell viability for each condition was depicted relative to that of mock-transfected cells at 24h. Significant differences were determined by t-test.
