# Supplementary material for: Interactome analysis of Bag-1 isoforms reveals novel interaction partners in endoplasmic reticulum-associated degradation
Source: PLoS One. 2021 Aug 24;16(8):e0256640. doi: 10.1371/journal.pone.0256640 (PMC8384158; doi:10.1371/journal.pone.0256640)
Supplement: S9 Fig — (DOCX) [file pone.0256640.s009.docx]

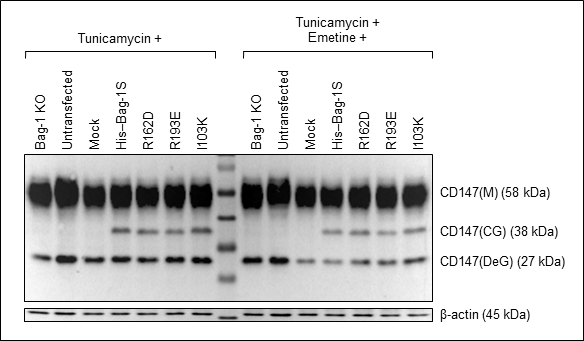


**Figure S9. Effect of double treatment with tunicamycin and emetine on CD147.** MCF-7 cells were treated with both glycosylation and translation inhibitor to follow the relation between CD147 glycosylation pattern and Bag-1 ex-pression (M: mature, CG: core glycosylated, DeG: deglycosylated).
